# Whole genome sequencing of a natural recombinant *Toxoplasma gondii *strain reveals chromosome sorting and local allelic variants

**DOI:** 10.1186/gb-2009-10-5-r53

**Published:** 2009-05-20

**Authors:** Irene Lindström Bontell, Neil Hall, Kevin E Ashelford, JP Dubey, Jon P Boyle, Johan Lindh, Judith E Smith

**Affiliations:** 1 Institute of Integrative and Comparative Biology, Clarendon Way, University of Leeds, Leeds, LS2 9JT, UK; 2 School of Biological Sciences, University of Liverpool, Crown Street, Liverpool, L69 7ZB, UK; 3 United States Department of Agriculture, Agricultural Research Service, Animal and Natural Resources Institute, Animal Parasitic Diseases Laboratory, Baltimore Avenue, Beltsville, MD 20705, USA; 4 Department of Biological Sciences, University of Pittsburgh, Fifth Avenue, Pittsburgh, PA 15260, USA; 5 Department of Parasitology, Mycology and Environmental Microbiology, Swedish Institute for Infectious Disease Control (SMI), Nobels väg, 171 82 Solna, Sweden; 6 Current address: Division of Clinical Microbiology, Department of Medicine, Karolinska Institutet, Alfred Nobels Allé, 141 86 Stockholm, Sweden

## Abstract

**Background:**

*Toxoplasma gondii *is a zoonotic parasite of global importance. In common with many protozoan parasites it has the capacity for sexual recombination, but current evidence suggests this is rarely employed. The global population structure is dominated by a small number of clonal genotypes, which exhibit biallelic variation and limited intralineage divergence. Little is known of the genotypes present in Africa despite the importance of AIDS-associated toxoplasmosis.

**Results:**

We here present extensive sequence analysis of eight isolates from Uganda, including the whole genome sequencing of a type II/III recombinant isolate, TgCkUg2. 454 sequencing gave 84% coverage across the approximate 61 Mb genome and over 70,000 single nucleotide polymorphisms (SNPs) were mapped against reference strains. TgCkUg2 was shown to contain entire chromosomes of either type II or type III origin, demonstrating chromosome sorting rather than intrachromosomal recombination. We mapped 1,252 novel polymorphisms and clusters of new SNPs within coding sequence implied selective pressure on a number of genes, including surface antigens and rhoptry proteins. Further sequencing of the remaining isolates, six type II and one type III strain, confirmed the presence of novel SNPs, suggesting these are local allelic variants within Ugandan type II strains. In mice, the type III isolate had parasite burdens at least 30-fold higher than type II isolates, while the recombinant strain had an intermediate burden.

**Conclusions:**

Our data demonstrate that recombination between clonal lineages does occur in nature but there is nevertheless close homology between African and North American isolates. The quantity of high confidence SNP data generated in this study and the availability of the putative parental strains to this natural recombinant provide an excellent basis for future studies of the genetic divergence and of genotype-phenotype relationships.

## Abstract 2

Extensive sequence analysis of eight Toxoplasma gondii isolates from Uganda has revealed chromosome sorting and local allelic variants.

## Background

*Toxoplasma gondii *is a ubiquitous protozoan parasite of medical and veterinary importance. It can be transmitted via vertical transmission, through carnivory and by ingestion of highly infectious oocysts excreted by the definitive felid hosts [1]. Despite its worldwide distribution, broad host range and multiple transmission routes, which give ample opportunities for strain partitioning and recombination, *T. gondii *has an unusual population structure dominated by a limited number of clonal lineages [2]. Experimental crosses have shown that a single mating opportunity between two strains in the definitive host can result in a multitude of new strains with altered phenotypic properties [3-6], yet this appears to be rare in nature and only three clonal strain types, called I, II and III, predominate across Europe and North America [7,8]. As data become available from wider geographical studies it is evident that higher levels of allelic variation and clonal expansion of non-archetypal lineages occur in South America [9-11]. While the global population structure may be more complex than previously thought, classification of strains into types I, II and III is still highly relevant in Europe, North America and possibly also in Africa [12].

The three lineages are believed to originate from a few crosses between closely related ancestral strains and generally show a biallelic single nucleotide polymorphism (SNP) pattern where, for any given chromosomal region, two out of the three strains share one allele while the third strain is different [7]. Only one chromosome (Ia) is virtually monomorphic among the three lineages and one chromosome (IV) is dominated by type III SNPs, while all the other chromosomes have a predominance of either type I or type II SNPs or display a chimeric SNP pattern [13]. The full genome sequences of one reference isolate from each of the three clonal lineages have been generated and are available through the *Toxoplasma *genome database, ToxoDB [14,15], and this detailed information has been used to reconstruct the deep evolutionary relationships between lineages [13,16]. Estimates of within lineage variation have also been made, with the focus on mapping the biogeographical distribution of strain haplotypes to infer patterns of dispersal and disease spread [12,17]. These studies are based on sequence analysis of selected loci from multiple strains, but no comparison has ever been made between two isolates from the same lineage at the genome level. In an environment where strains from a single lineage dominate, it becomes important to estimate the level of allelic variation as recombination may mainly be between strains of the same type.

Recent studies have found evidence of clonal types I, II and III in Africa [18,19], a continent with a wide range of diverse habitats and, like South America, many felid species. Our previous study [19] identified mixed infections in five out of twenty free-range chickens (*Gallus domesticus*). The presence of multiple strains in a single intermediate host increases the likelihood of recombination between genotypes. Initial analysis of isolates from this source led to the identification of a putative natural recombinant strain. In this study, we report the whole genome sequencing of this isolate, TgCkUg2, a recombinant between type II and III. Alignment with the reference genomes for Me49 (type II) and VEG (type III), revealed which parts of the genome were inherited from the respective parental strains, and, furthermore, allowed us to look for intralineage divergence and discover new polymorphisms. Comparisons with additional isolates from the same source, one with type III and six with type II alleles, enabled detection of local allelic variants and preliminary genotype-phenotype associations. This is the first whole genome sequencing of a recombinant *T. gondii *strain and the quality of information generated and availability of the putative parental strains to this natural recombinant provide an excellent basis for a better understanding of the gene combinations responsible for virulence and successful transmission of *T. gondii*.

## Results

### Sequencing and SNP mapping in TgCkUg2

Preliminary genotyping led to the conclusion that one of our eight Ugandan isolates contained loci typical of both type II and type III strains and was therefore likely to be a natural recombinant. To gain more information on the nature of the recombination event and on the relationship between the reference strain types and this isolate, TgCkUg2 was subjected to whole genome sequencing and SNP mapping using the 454 Life Sciences platform. Three runs were performed, generating approximately fourfold coverage. We assembled 673,878 reads into 67,013 contigs, ranging from 95 to 12,769 bp with an average length of 773 bp. The contigs were aligned against the complete genome sequence of the Me49 reference strain using version 4.3 of the Toxoplasma database [14] and found to span 51.84 Mb, corresponding to a genome coverage of 84% (full details of data deposition are given in Materials and methods). There was no particular bias between the 14 chromosomes in terms of the read density or contig coverage. The data generated for all chromosomes are summarised in Table 1.

**Table 1:** Summary of 454 whole genome sequencing output and SNP identification of a type II/III recombinant *T. gondii *strain

Number	Chromosome length (bp)	Number of reads*	Total contig length (bp)	Coverage by contigs	Average reads/kb	Type II SNPs* (Green^†^)	Type III SNPs^‡ ^(Blue^†^)	Novel SNPs^§ ^(Orange^†^)	Major SNP^¶ ^density/kb	Minor SNP^¥ ^density/kb
Ia	1,896,408	20,649	1,585,140	83.59%	13.03	2	128	7	0.067	0.005
Ib	1,956,324	20,583	1,639,876	83.82%	12.55	1	1,483	10	0.758	0.006
II	2,302,931	24,968	1,939,495	84.22%	12.87	4,370	73	52	1.898	0.054
III	2,470,845	26,771	2,060,909	83.41%	12.99	27	4,224	73	1.710	0.040
IV	2,576,468	27,510	2,150,897	83.48%	12.79	4,731	176	60	1.836	0.092
V	3,147,601	33,619	2,582,080	82.03%	13.02	54	5,725	26	1.819	0.025
VI	3,600,655	39,723	3,042,491	84.50%	13.06	1,985	168	99	0.551	0.074
VIIa	4,502,211	48,365	3,797,608	84.35%	12.74	8,663	79	56	1.924	0.030
VIIb	5,023,822	53,768	4,231,651	84.23%	12.71	62	5,530	41	1.101	0.021
VIII	6,923,375	75,308	5,851,305	84.52%	12.87	123	6,775	158	0.979	0.041
IX	6,384,456	72,298	5,337,365	83.60%	13.55	8,119	325	269	1.272	0.093
X	7,418,475	85,205	6,298,377	84.90%	13.53	13,459	236	209	1.814	0.060
XI	6,570,290	70,653	5,549,592	84.46%	12.73	49	60	117	-	0.034
XII	6,871,637	74,458	5,770,139	83.97%	12.90	58	4,809	75	0.700	0.019
Total	61,645,498	673,878	51,836,925	84.09%	13.00	41,703	29,791	1,252	1.264	0.042

**Figure 1:**
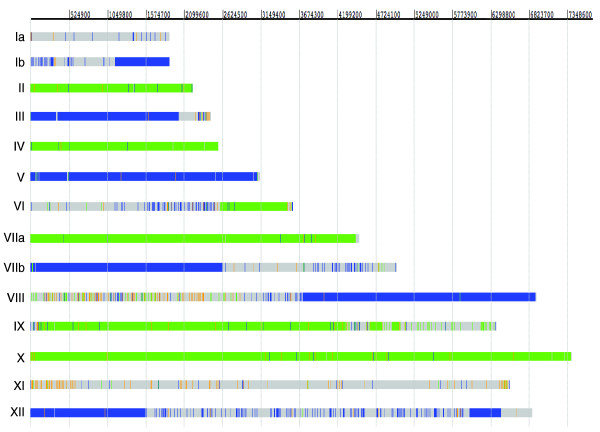
SNP distribution in TgCkUg2. The genomic sequence of TgCkUg2 was aligned with the sequences of Me49 and VEG [14]; the SNP distribution over the 14 chromosomes is shown above. Green SNPs denote a type II background, where TgCkUg2 is identical to Me49, but different from VEG. Blue SNPs denote a type III background, where TgCkUg2 is identical to VEG, but different from Me49. Orange indicates novel SNPs where TgCkUg2 is different from both reference strains. Grey areas are devoid of SNPs between these strains; genotypes II and III are highly similar in these regions.

**Figure 2:**
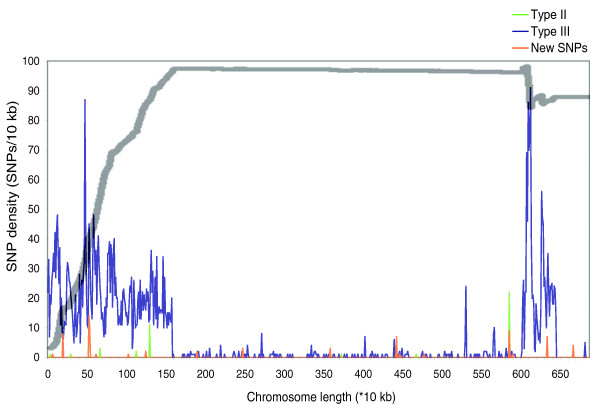
Matching of type I SNP dominance and regions with a low SNP density in TgCkUg2. Comparison of SNP patterns in TgCkUg2 with those in the three sequenced reference genomes, GT1 (I), Me49 (II) and VEG (III) on chromosome XII. The underlying graph depicts the SNPs in TgCkUg2 relative to the type II and III reference strains (green and blue, respectively) as well as those unique to TgCkUg2 (orange). In TgCkUg2, chromosome XII was derived from the type III parent (as shown by a predominance of TgCkUg2 SNPs that matched the reference type III at that position), but had large regions with a very low SNP content. To correlate these regions of high and low polymorphism content with existing polymorphism data, all type I, II and III SNPs derived from the reference genome sequences were obtained. For each identified SNP, a running sum was computed across the chromosome as follows: +0 for a type I SNP, +1 for a type II SNP, and -1 for a type III SNP. This running sum was then plotted against the position in the genome of that SNP, creating the grey line shown. This shows that for the first 1.5 Mb of chromosome XII, type II SNPs predominate in the reference strains (types I, II and III, as indicated by the rising line), but at these positions TgCkUg2 has the type III allele (as indicated by the blue line). From approximately 1.5 Mb to 6 Mb, the chromosome is dominated by type I SNPs in the reference strains (as indicated by the straight grey line) and correspondingly there are very few polymorphisms between TgCkUg2 and the reference strains (similar maps for all 14 chromosomes can be found in Additional data file 2).

*SNPs denoting a type II background. TgCkUg2 is identical to Me49, but different from VEG. ^†^The colors correspond to the colours used for the respective SNP types in Figures 1 and 2 and Additional data files 1 and 2. ^‡^SNPs denoting a type III background. TgCkUg2 is identical to VEG, but different from Me49. ^§^SNPs where TgCkUg2 has a novel allele, different from both Me49 and VEG. ^¶^The predominant SNP type, corresponding to the chromosomal background type. ^¥^SNPs where TgCkUg2 differs from the background type, including novel SNPs.

To determine the relative contribution of type II and type III regions to the recombinant isolate, the genome of TgCkUg2 was compared to Me49 (II) and VEG (III). SNPs were defined based on 100% concordance over a minimum of three reads, of which at least one was in the forward direction and one in the reverse. The total number of unambiguous SNPs identified using these stringent criteria and excluding repeat regions was 72,746, which corresponds to about a quarter of the > 300,000 known polymorphisms between Me49 and VEG. Most SNPs were mapped against either Me49 or VEG, so that TgCkUg2 had the same allele as one of the reference strains, reflecting the origin of this chromosomal region. In addition, 1,252 novel polymorphisms were found where TgCkUg2 was different from both Me49 and VEG. The distribution of SNPs called against the two reference strains was highly disproportionate (Figure 1; Additional data file 1) and indicated the genotype of each chromosome. Chromosomes II, IV, VI, VIIa, IX and X were inherited from a type II-like strain while chromosomes Ia, Ib, III, V, VIIb, VIII and XII originated from the type III-like parent.

Due to the paucity of SNPs between types II and III on chromosome XI, it was difficult to derive its source of origin. In total, 226 SNPs were called over its full length of > 6.5 Mb, which averages one SNP per 29 kb. Most of these (117 SNPs) were unique to TgCkUg2, while 49 positions were identical to Me49 and 60 to VEG. There was no obvious clustering of SNPs according to strain on this chromosome and, therefore, no evidence of chromosomal recombination. In total, across all chromosomes, there was a nearly equal contribution from both parental strains to the genome of TgCkUg2, which is consistent with a single sexual reproduction event. Six type II and seven type III chromosomes encompassing 26.8 and 28.3 Mb, respectively, were found, plus one chromosome that might derive from either parent.

Seven chromosomes (Ib, III, VI, VIIb, VIII, IX and XII) showed dramatic changes in the density of their predominant SNP type across their length (Figure 1; Additional data file 1). The predominant type, or 'major SNP', matches the parental allele and corresponds to the divergence between lineages II and III, while the term 'minor SNP' is used for SNPs that do not match the background type of the chromosome. Absence of major SNPs signifies a high level of similarity between types II and III, which corresponds to regions dominated by type I SNPs in the comparison between the three reference strains (where biallelic SNPs are named by the diverging genotype [13]).

Data from the three reference type strains were used to map the relative abundance of type I, II and III SNPs across the parasite genome. Comparison of SNPs from the recombinant strain against this distribution demonstrated that all regions without major SNPs in TgCkUg2 corresponded to the regions dominated by type I SNPs (Figure 2; Additional data file 2). The close matching of these independently retrieved data sets provides strong evidence that TgCkUg2 is the progeny of a cross between modern type II and III strains, where chromosome sorting was the main mechanism of recombination.

### The apicoplast

Most of the apicoplast genome (> 71%) was covered by a single large contig of 25 kb. The read density was considerably higher than the average read density for the chromosomal contigs: 121.5 reads/kb for the apicoplast compared with 12.55 to 13.55 reads/kb for the chromosomal regions (Table 1). The unbiased mechanism of 454 sequencing results in automatic quantification of amplified regions [20], and the higher read density thus implies an average apicoplast genome copy number of nine or ten. This result is slightly higher than the 5 to 7 copies reported initially [21,22], but lower than the > 25 copies suggested later [23], which could be due to inherent differences between strains or methodological differences.

The apicoplast sequence currently available in ToxoDB is from RH, a type I strain. Alignment of the apicoplast genomes of TgCkUg2 and RH resulted in 23 SNP calls over 25,069 bp of sequence. The sequence surrounding each of these SNPs was BLASTed against the sequence data from Me49, VEG and GT1 in the NCBI Trace Archive. Out of 23 high-confidence SNPs detected between TgCkUg2 and RH, all positions were identical in TgCkUg2, Me49 and VEG, while the comparison to GT1 showed three discrepancies. The apicoplast is inherited from the macrogamete in a cross [24], but due to the high level of similarity between types II and III and the fragmented nature of the data in the NCBI Trace archive, it was not possible to ascertain the maternal inheritance of the recombinant strain.

### Novel SNPs

**Figure 3:**
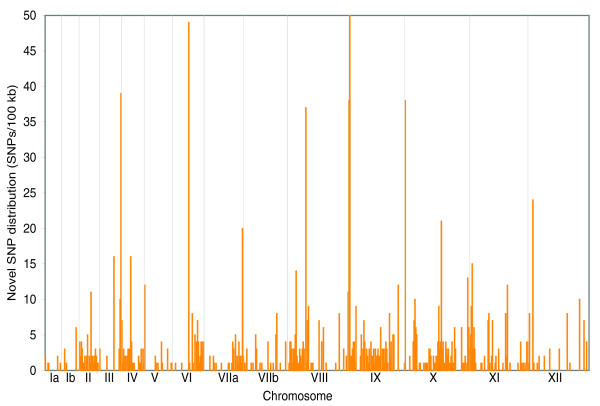
Location and density of unique SNPs in TgCkUg2. The graph shows the number of SNPs per 100 kb, where TgCkUg2 had a different allele compared with Me49 and VEG. New SNPs were distributed across the whole genome, but very high densities were found near the telomeres of chromosomes III, VIIa, IX and X, but also in central regions of chromosomes VI and VIII. These mutation hot-spots were mostly located in intergenic regions, but also caused a high number of mutations in the genes for hypothetical proteins 2.m00067, 42.m07434 and the rhoptry antigen ROP5 (551.m00238).

In the alignments of the TgCkUg2 genome with Me49 and VEG, 1,252 positions were found where the two reference strains were identical but the Ugandan strain TgCkUg2 had a different allele. However, based on the SNP discovery rate with the coverage and cut-off criteria we used, the real density of novel SNPs is likely to be around four times higher. The new SNPs were dispersed across all chromosomes, and they occurred at an average frequency of one per 50 kb, but at a higher frequency in the subtelomeric regions of chromosomes (terminal 10%). In total, 38.1% of novel SNPs were found in these regions compared with 21.4% of all SNPs, and this difference was highly significant (*P *< 0.001, chi-square test). Several chromosomes had one, or a few, clusters with a high level of new mutations and smaller clusters were found on all chromosomes except Ia. The highest concentrations of new SNPs were found in the subtelomeric regions of chromosomes III, IX and X, and in more central regions of VI and VIII (Figure 3). These novel SNPs occasionally coincided with the allele found in the type I reference strain but most are likely to be the result of new mutations. However, within a short region encompassing 103 bp on chromosome IV, 16 SNPs were found where TgCkUg2, and several of the other Ugandan type II strains, were similar to GT1, but different from VEG and Me49 (Table 2). This similarity only applied to a short region near the chromosomal end and could be a remnant of an earlier recombination event.

**Table 2:** Polymorphisms on chromosome IV, positions 8,805 to 8,907, where TgCkUg2 and several Ugandan type II strains shared allelic variants with GT1 (type I)

Locus	IV-8															
**Me49 (II)**	**T**	**C**	**T**	**A**	**T**	**T**	**G**	**G**	**A**	**G**	**G**	**G**	**A**	**A**	**G**	**T**
**VEG (III)**	*	*	*	*	*	*	*	*	*	*	*	*	*	*	*	*
TgCkUg6	*	*	*	*	*	*	*	*	*	*	*	*	*	*	*	*
TgCkUg5	*	*	*	*	*	*	*	*	*	*	*	*	*	*	*	*
TgCkUg9	*	*	*	*	*	*	*	*	*	*	*	*	*	*	*	*
TgCkUg7	*	*	A	T	C	C	C	*	*	T	*	*	G	C	C	*
TgCkUg1	C	A	A	T	C	C	C	C	C	T	A	A	G	C	C	A
TgCkUg2	C	A	A	T	C	C	C	C	C	T	A	A	G	C	C	A
TgCkUg3	C	A	A	T	C	C	C	C	C	T	A	A	G	C	C	A
TgCkUg8	C	A	A	T	C	C	C	C	C	T	A	A	G	C	C	A
**GT1 (I)**	**C**	**A**	**A**	**T**	**C**	**C**	**C**	**C**	**C**	**T**	**A**	**A**	**G**	**C**	**C**	**A**

Similarities to the Me49 sequence are indicated by an asterisk.

Novel SNPs were assigned as non-coding, synonymous or non-synonymous based on gene annotations for Me49 from the Toxoplasma genome database [14]. Most of the novel polymorphisms were non-coding mutations in intergenic or intronic regions, but among the coding SNPs there were twice as many non-synonymous as synonymous mutations, 111 and 55, respectively. Fifteen genes were identified that had at least two novel SNPs in the coding sequence of TgCkUg2 and these are listed in Table 3. Nine of these had a predominance of non-synonymous SNPs and three genes contained six or more mutations that resulted in amino acid substitutions: genes 2.m00067 and 49.m03279, which are currently annotated as hypothetical proteins in ToxoDB, and gene 551.m00238 on chromosome XII, which encodes the secreted rhoptry kinase ROP5.

**Table 3:** Genes with more than two novel SNPs in the coding region of TgCkUg2

Gene ID (v4.3)*		Novel SNPs in TgCkUg2		SNPs between the three lineages^†^		
				
	Number	Synonymous	Non-synonymous	Synonymous	Non-synonymous	Protein description
641.m01562	IV	1	1	12	86	SRS16B
641.m02553	IV	1	1	6	1	WD-40 repeat protein, putative
						
49.m03276	VI	1	1	0	4	ROP29
49.m03279	VI	2	8	1	13	Hypothetical
49.m03372	VI	1	1	13	3	Long chain fatty acid CoA ligase
						
55.m04829	VIIb	1	3	1	2	SRS26A
44.m02583	VIII	0	2	9	11	Hypothetical
44.m05903	VIII	0	2	7	8	Hypothetical
57.m01765	IX	0	2	143	231	Protein kinase domain containing
						
2.m00067	IX	3	7	0	0	Hypothetical
57.m01732	IX	0	2	7	8	Hypothetical
80.m02252	IX	1	1	4	2	Phosphoenolpyruvate carboxykinase, putative
						
42.m07434	X	0	2	0	0	Hypothetical
551.m00238	XII	3	6	8	44	ROP5
65.m00001	XII	4	0	9	6	NTPase I

*Since the comparison was made against the annotation of Me49 in v4.3 of ToxoDB, these gene IDs are used throughout. These remain searchable in the current annotation (v 5.0). ^†^Data from ToxoDB showing the total number of SNPs between GT1, Me49 and VEG, in order to put the number of novel SNPs into context.

In order to detect genes under selection, we used the whole genome sequences from Me49, VEG and TgCkUg2 and performed maximum likelihood pairwise comparisons between all genes to calculate the ratio of non-synonymous to synonymous mutations (dN/dS). This was followed by a likelihood ratio test to select genes that had a dN/dS ratio significantly (*P *< 0.05) higher than one [25]. Using these criteria, evidence for selection was detected for 46 genes (Additional data file 3). These candidates included genes encoding four dense granule proteins, GRA3 (42.m00013), GRA6 (63.m00002), GRA7 (20.m00005) and GRA8 (52.m00002), the rhoptry kinase family protein ROP4/7 (83.m02145) and the bradyzoite surface protein SRS16B (641.m01562), previously identified in the analysis of novel SNP clusters (Table 3). A subset of 16 genes had very high dN/dS values, indicating that they may be under positive selection in TgCkUg2 (Table 4). These included the genes encoding GRA3 and ROP4/7 and 551.m00237, a gene immediately adjacent to that encoding ROP5. One gene (42.m07434) located on chromosome X exhibited significant divergence between TgCkUg2 and its chromosomal background genotype. This is currently annotated as a hypothetical protein and nothing is known about its function or localization.

**Table 4:** Genes under selection identified by dN/dS analysis in TgCkUg2

Chromosome (type*)	Comparator	Gene ID (v4.3)	Protein description	dN/dS ratio^†^	*P*-value
Ia (III)	Me49 (II)	83.m02145	Rhoptry kinase ROP4/ROP7	Infinity	0.025 <*P *< 0.050
IV (II)	VEG (III)	641.m01516	Hypothetical	Infinity	0.005 <*P *< 0.010
V (III)	Me49 (II)	39.m00623	Proline-rich protein	Infinity	0.010 <*P *< 0.025
V (III)	Me49 (II)	31.m01816	Iron-sulfur cluster assembly accessory protein, putative	Infinity	0.010 <*P *< 0.025
					
V (III)	Me49 (II)	76.m01544	Hypothetical	Infinity	0.010 <*P *< 0.025
VI (II)	VEG (III)	49.m03376	Hypothetical	Infinity	0.025 <*P *< 0.050
VI (II)	VEG (III)	49.m03382	Hypothetical	Infinity	0.010 <*P *< 0.025
VI (II)	VEG (III)	49.m03431	Hypothetical	Infinity	0.010 <*P *< 0.025
VIII (III)	Me49 (II)	59.m07776	Hypothetical	Infinity	0.025 <*P *< 0.050
VIII (III)	Me49 (II)	59.m03361	Transporter, major facilitator family domain containing	4.325	0.010 <*P *< 0.025
					
X (II)	Me49 (II)	42.m07434	Hypothetical	Infinity	0.010 <*P *< 0.025
X (II)	VEG (III)	42.m03570	LytB domain-containing protein	Infinity	0.025 <*P *< 0.050
X (II)	VEG (III)	42.m00013	GRA3	Infinity	0.010 <*P *< 0.025
X (II)	VEG (III)	46.m02909	Hypothetical	Infinity	0.025 <*P *< 0.050
XII (III)	Me49 (II)	551.m00237	Hypothetical	Infinity	0.025 <*P *< 0.050
XII (III)	Me49 (II)	145.m00337	Hypothetical	6.527	0.010 <*P *< 0.025

*The chromosomal background genotype. ^†^The number of non-synonymous SNPs divided by the number of synonymous SNPs. In most cases, no synonymous SNPs were found, and this rate approaches infinity.

### Estimated divergence of African and reference isolates

SNP data from TgCkUg2 were used to estimate the age of the most recent common ancestor (MRCA) of the Ugandan types II and III (UgII and UgIII) and the reference strains of the respective types. Six type II chromosomes of this recombinant strain were used for the Me49/UgII calculations and seven chromosomes of type III origin were used to estimate the VEG/UgIII split. Calculations are shown for all chromosomes separately as well as for the full type II and type III sequences using two different approaches (Tables 5 and 6).

**Table 5:** Calculation of the MRCA of Ugandan type II and III isolates and reference strains based on the intron mutation rate

Chromosome	Intron length* (bp)	Minor SNPs in introns	MRCA Me49/UgII (years)	MRCA VEG/UgIII (years)
Type II				
II	89,003	34	19,691	
IV	85,471	58	34,979	
VI	152,385	65	21,987	
VIIa	198,902	28	7,256	
IX	266,876	117	22,598	
X	334,181	79	12,186	
Total II	1,126,818	381	17,429	
				
Type III				
Ia	80,522	2		1,280
Ib	85,805	8		4,806
III	82,660	9		5,612
V	119,536	15		6,468
VIIb	220,517	39		9,116
VIII	337,557	78		11,911
XII	352,605	78		11,403
Total III	1,279,202	229		9,228

The divergence between Ugandan and reference strains were calculated based on the intron mutation rate 1.94 × 10^-8 ^[26]. A more comprehensive version is provided in Additional data file 4. *Length of intronic regions. MRCA, most recent common ancestor.

**Table 6:** Calculation of the MRCA of Ugandan type II and III isolates and reference strains based on their relationship to the divergence between Me49 and VEG

Chromosome	Length* (bp)	Minor SNPs	Major SNPs	MRCA Me49/UgII (years)	MRCA VEG/UgIII (years)
Type II					
II	2,302,931	125	4,370	4,291	
IV	2,576,468	236	4,731	7,483	
VI > 2.6 Mb	1,000,655	70	1,913	5,489	
VIIa	4,502,211	135	8,663	2,338	
IX 0.5-4 Mb	3,500,000	205	6,349	4,843	
X	7,418,475	445	13,459	4,960	
Total II	21,300,740	1,216	39,845	4,578	
					
Type III					
Ib > 1.2 Mb	756,324	6	1,375		655
III < 1.9 Mb	1,900,000	33	3,953		1,252
V	3,147,601	80	5,725		2,096
VIIb < 2.5 Mb	2,500,000	43	5,069		1,272
VIII > 4 Mb	2,923,375	49	6,268		1,173
XII < 1.5 Mb	1,500,000	69	3,259		3,176
Total III	12,727,300	280	25,648		1,638

The divergence between Ugandan and reference strains were calculated based on their relationship to the divergence between Me49 and VEG, with the MRCA estimated to be at 150,000 years ago [27]. A more comprehensive version is provided in Additional data file 4. *Length of regions with a major SNP type. MRCA, most recent common ancestor.

The estimated *T. gondii *intron mutation rate of 1.94 × 10^-8 ^mutations per nucleotide per year [26] was applied to minor SNPs found in intronic regions across the genome. This was achieved by retrieving all SNPs where TgCkUg2 was different from Me49 within the introns of type II chromosomes (II, IV, VI, VIIa, IX and X), and similarly all SNPs where TgCkUg2 differed from VEG for type III chromosomes (Ia, Ib, III, V, VIIb, VIII and XII). In total, the type II intronic regions contained 381 minor SNPs over 1.13 Mb, which gives an estimate of 17,400 years for the MRCA of UgII and Me49. The type III regions contained 229 SNPs over 1.28 Mb, giving an estimate for the divergence of UgIII and VEG of 9,200 years. Substantial variation was found between chromosomes from the same lineage; however, all type II chromosomes except VIIa gave earlier divergence time estimates than chromosomes of type III.

The second method related data on major and minor SNPs, where major SNPs were assumed to represent the divergence between types II and III at the nucleotide level based on an estimated MRCA at 150,000 years ago [27], while minor SNPs were assumed to represent the intralineage divergence between Ugandan and reference strains. Regions dominated by type I SNPs were excluded from this analysis since they do not contain a major SNP type. These calculations resulted in divergence time estimates, which were considerably more recent; 4,600 years for UgII/Me49 and 1,600 years for UgIII/VEG. The overall genomic mutation rate was calculated by a weighted average of the type II and III regions and estimated to be approximately 1.28 × 10^-8 ^mutations per nucleotide per year, which corresponds to 66% of the rate calculated for intronic regions.

Finally, an estimate of the age of the MRCA of all strains was calculated based on major and minor SNPs in intronic regions using the results obtained by application of the intron mutation rate. These estimates were considerably higher than the proposed 150,000 years, suggesting a MRCA about 10^6 ^years ago, which is similar to the timing proposed for the divergence of South American strains [8]. The data used for these calculations are provided in Additional data file 4.

### Relationships between Ugandan isolates

In addition to TgCkUg2, one type III strain, TgCkUg6, and six type II strains, TgCkUg1, 3, 5, 7, 8 and 9, were isolated from Uganda. We generated and compared sequence data over > 20 kb across 34 loci (Additional data file 5) distributed across the genome to investigate the genetic relatedness among Ugandan *T. gondii *strains. These loci included known polymorphic genes such as those encoding toxofilin and ROP18, microsatellites and intronic regions. A high level of sequence homology was seen between the novel isolates from Uganda and the reference strains, which originate from North America. The type III strain, TgCkUg6, was very closely related to the type III reference strain VEG as well as the type III regions of TgCkUg2. In comparison to VEG, TgCkUg6 had 39 SNPs over 20.9 kb and most of these SNPs were concentrated in two loci: II-4 (10 SNPs over 598 bp) and VI-13 (18 SNPs over 368 bp). Apart from these regions the sequence identity between the type III strains was > 99.9%. Locus II-4 consisted of non-coding subtelomeric sequence on chromosome II, where TgCkUg6 shared some alleles with strains of genotype II (including Me49). The second locus, VI-13, included 220 bp of the coding sequence of the surface protein SRS22H (49.m03110), where several new, non-synonymous SNPs were found for TgCkUg6, TgCkUg2 and three of the Ugandan type II strains (Table 7).

**Table 7:** Local allelic variants in Ugandan *T. gondii *strains leading to amino acid changes in two surface proteins (SRSs) and one rhoptry protein (toxofilin)

Locus amino acid position	SRS22H (VI-13)										Toxofilin			SRS16B
			
	111	113	138	139	140	141	143	144	146	150	147	168	176	77
**Me49 (II)**	**E**	**E**	**K**	**P**	**S**	**A**	**H**	**R**	**T**	**D**	**L**	**E**	**K**	**A**
TgCkUg5	*	G	*	G	*	*	*	*	*	*	*	*	R	*
TgCkUg9	*	*	*	*	*	*	*	*	*	V	*	D	R	*
TgCkUg7	*	*	*	*	*	*	*	*	*	*	Q	D	R	E
TgCkUg1	*	*	*	*	*	*	*	*	*	*	Q	*	*	E
TgCkUg8	*	*	*	*	*	*	*	*	*	*	Q	*	*	E
TgCkUg3	*	*	*	*	*	*	*	*	*	V	Q	*	*	E
TgCkUg2	D	G	T	G	T	G	R	*	P	V	Q	*	*	E
TgCkUg6	D	G	N	G	*	G	R	S	P	V	E	*	*	T
**VEG (III)**	*	*	*	*	*	*	*	*	*	*	**E**	*	*	**T**
**GT1 (I)**	*	**G**	**S**	**A**	**T**	**E**	**R**	**S**	**D**	**G**	**Q**	*	**R**	**A**

Similarities to the reference sequence for Me49 are indicated by an asterisk.

The Ugandan type II isolates, including the type II regions of TgCkUg2, were closely related to Me49 (> 99.5% sequence identity), but with some allelic variation. The new SNPs were largely concentrated at a few loci and many were shared among Ugandan isolates, suggesting that these are local allelic variants. Interestingly, most new SNPs found within genes, including those encoding toxofilin (33.m02185) and SRS16B (641.m01562), resulted in amino acid changes, suggesting they may be under selection. Based on these variants, it was possible to resolve that TgCkUg5 and TgCkUg9 were the isolates most similar to Me49 and that TgCkUg3 was the strain most similar to the type II component of the recombinant TgCkUg2.

This complementary sequencing confirmed the assignment of TgCkUg2 chromosomes according to 454 SNP analyses, but discovered possible chromosomal recombination events in two type I dominated regions. Chromosome VIII was identified as derived from type III based on the major SNP density in the second half of the chromosome. However, for loci VIII-19 and VIII-20 (located at 0.9 and 2.1 Mb) TgCkUg2 was more similar to Me49 than VEG and even contained four allelic variants that were also present in the Ugandan type II isolate TgCkUg8, while locus VIII-21 at 5.8 Mb identified TgCkUg2 as a type III strain (Table 8). Similarly, comparison of TgCkUg2 and TgCkUg6 sequence for the VI-13 locus, located around 0.3 Mb, indicated the presence of a type III region in the otherwise type II derived chromosome VI. These results provide indications of chromosomal recombination in TgCkUg2, but the limited extension of the SNP peaks at these locations (Additional data file 1) suggest gene conversions rather than homologous cross-over events.

**Table 8:** Indication of intrachromosomal recombination in TgCkUg2

Locus position	VIII-19 0.9 Mb							VIII-20 2.1 Mb				VIII-21 5.8 Mb		
**Me49 (II)**	**A**	**T**	**G**	**C**	**T**	**T**	**T**	**C**	**T**	**G**	**A**	**G**	**C**	**G**
TgCkUg5	*	*	*	*	*	*	*	*	*	*	*	*	*	*
TgCkUg8	*	*	*	*	A	C	*	T	*	A	*	*	*	*
TgCkUg2	*	*	*	*	A	C	*	T	*	A	*	A	A	T
TgCkUg6	T	*	C	G	*	*	C	*	C	*	T	A	A	T
**VEG (III)**	**T**	**C**	**C**	**G**	*	*	**C**	*	**C**	*	**T**	**A**	**A**	**T**

For loci VIII-19 and VIII-20 located around 0.9 and 2.1 Mb on chromosome VIII, TgCkUg2 is type II-like and shares some local allelic variants with TgCkUg8. Later on the chromosome, TgCkUg2 is type III-like, as shown by the VIII-21 locus and the major SNP type (Figure 1; Additional data file 1).

### Phenotype of TgCkUg2 and clonal Ugandan isolates

**Figure 4:**
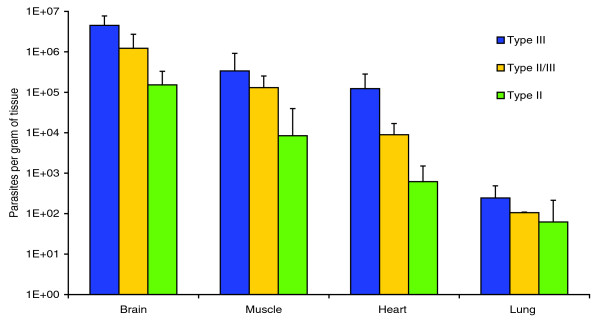
Parasite density in mouse tissues. The parasite burden in mice (number of *T. gondii *per gram) was determined by quantitative PCR and the graph shows the average values plus standard deviation for one type III strain (three mice), one type II/III strain (two mice) and six type II strains (17 mice). The type III strain (TgCkUg6) caused the highest burden, followed by the recombinant strain (TgCkUg2), while the six type II strains (TgCkUg1, 3, 5, 7, 8, 9) had significantly lower densities. In all mice, the brain was the most heavily infected tissue.

None of the Ugandan isolates caused morbidity or mortality in mice and could therefore be classified as avirulent. Quantitative PCR (Q-PCR) of parasite burden in mice used for isolation revealed major differences between strain types (Figure 4). The type III strain TgCkUg6 produced high tissue burdens compared with the Ugandan type II strains, and this difference was significant for brain (*P *< 0.001), heart (*P *< 0.002) and muscle (*P *< 0.02, *t*-test). The type II/III strain (TgCkUg2) caused an intermediate parasite burden for all organs. In brain tissue, the average density estimated via Q-PCR was 4.5 × 10^6 ^parasites per gram for type III, 1.2 × 10^6 ^for the recombinant, and 1.5 × 10^5 ^for the six type II strains. In heart tissue the mean values for parasite density were 1.2 × 10^5 ^(III), 8.9 × 10^3 ^(II/III) and 6.2 × 10^2 ^(II) parasites per gram. The parasite burden caused by the recombinant strain was significantly higher than type II strains (*P *< 0.003 for brain, heart and muscle), while the difference between TgCkUg2 and TgCkUg6 did not reach significance. In all mice, the brain was the most heavily infected organ (*P *≤ 0.05, paired *t*-test), and, on average, had more than tenfold higher parasite density than skeletal muscle, 100 times more than heart muscle and 1,000 times more than lung tissue.

**Figure 5:**
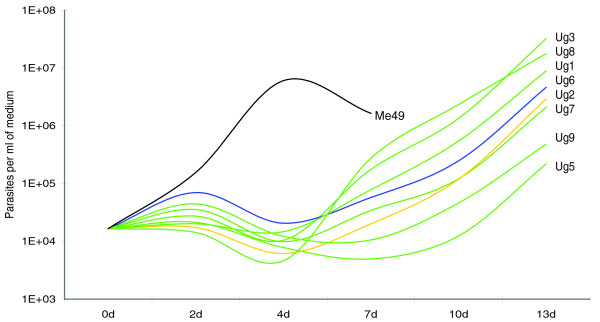
Number of extracellular parasites per milliliter of medium during growth in cell culture. Q-PCR quantification of free parasites in cell culture (number of *T. gondii*/ml medium) at five different time points (0 to 13 days after inoculation) revealed an extended lag phase in all the Ugandan isolates compared with Me49. TgCkUg6 (blue) and TgCkUg2 (orange) had intermediate growth rates, and the fastest growing Ugandan strains were TgCkUg1, 3 and 8, while TgCkUg5 and 9 were extremely slow growing and never reached high numbers of free parasites. The difference between the Ugandan isolates and Me49 is probably due to their recent introduction in cell culture, but the difference between the Ugandan type II strains may have a genetic component. Interestingly, TgCkUg5 and 9 were the strains with the lowest frequency of local allelic variants.

Parasite isolates were introduced into culture in human fibroblasts and growth characteristics were assessed by Q-PCR at passage eight. The growth of all Ugandan isolates was slow in cell culture in comparison to the reference strain Me49 (Figure 5). This difference was chiefly due to a prolonged lag phase of between 4 and 7 days, which preceded the phase of exponential growth. There was considerable variation between the type II strains, while the type III and the recombinant strain both had intermediate growth rates *in vitro*. Among the type II isolates, TgCkUg5 and TgCkUg9 were the slowest growing and never reached the parasite density levels of the other strains. Figure 5 shows the data for a single passage, but the slower growth of these two strains was consistently observed over several months.

## Discussion

Whole genome sequencing of a natural recombinant *T. gondii *strain revealed a nearly equal contribution of type II and III alleles, and demonstrated that it is likely to have arisen through a single recombination event between two parental strains. Most chromosomes appeared to have been inherited in their entirety from one parental strain, and the background genotype could easily be deduced from the SNP density. To our knowledge, the genetic combination found in TgCkUg2 has not been detected through multi-locus genotyping elsewhere (C Su, personal communication) nor been observed in progeny from laboratory crosses [28]. Even though novel SNPs were observed in the Ugandan strains, the whole genome sequence of TgCkUg2 showed a high level of sequence identity to the relevant reference strain, either Me49 or VEG for any given region. Certain chromosomal regions had very low SNP densities and these immediately appeared to coincide with the predominance of type I SNPs [13], which was confirmed by detailed comparisons across the genome (Additional data file 2). The sequencing of this recombinant isolate does not provide evidence of exotic or ancient strains in Uganda, but demonstrates sexual recombination through chromosome sorting between two strains that are closely related to the clonal lineages predominant in Europe and North America.

Sexual recombination between different strains of *T. gondii *appears to be an important evolutionary force, but has been assumed to be a rare event in nature [7]. For recombination to occur more than one genotype must be present in the environment and these must be ingested by the definitive host within a short time frame. The most plausible way for this to happen is for a cat to eat a host animal that is infected by two different strains, but the strong immune response elicited by the primary infection may prohibit the establishment of a second infection in some host species [29,30]. However, the possibility that multiple infections may occur in certain hosts has been proven in laboratory mice [31-33] and is also reported in naturally acquired infections in humans and animals [34-36], including the current strains from Uganda [19]. However, the relative abundance of the different strains in the host tissues may vary considerably and if one strain predominates, self-fertilization is likely to be more frequent than recombination between strains. In our Ugandan strains, we found that the difference in parasite burden in mice was up to 1,000-fold. In the event of multiple infections it is likely that only the most abundant strain would prevail. Indeed, some of our current isolates were obtained from mice inoculated with tissue containing more than one *T. gondii *genotype [19] but following passage in mice only one strain was recovered. Although there appear to be many obstacles to a successful recombination event, it is likely to be more frequent in environments where multiple parasite strains circulate within an area with close contact between cats and intermediate hosts [35,37].

Analysis of progeny from three experimental crosses (two II × III [5,6] and one I × III [38]) have shown that strains of different genotypes readily recombine to create progeny with predicted levels of exchange. On average, recombinant progeny had three to four recombinant chromosomes; however, one strain had as many as 10 recombinant chromosomes and 5 out of 75 strains (6.7%) had none [3,28]. Investigations of many more natural recombinant isolates are required to discover which gene combinations generate successful parasites and whether relative absence of hybrid chromosomes is beneficial and more common in nature than expected from the frequencies reported in the experimental crosses [3,5,6,38].

Although there was limited genetic divergence between the Ugandan strains and the type reference strains, SNPs against the background type were useful for estimation of the intralineage divergence and for detection of genes under selection. Divergence time estimates rely on the assumption that the mutation rate in a species remains relatively constant over time [39,40]. Without fossil records, calibration of the clock is difficult and in an organism with a complex life cycle, calculation of the mutation rate may be difficult. It is perhaps not surprising that the two methods, which use different data sets and start with different assumptions, give diverging time estimates. The relative timing, however, consistently indicated that the Ugandan type II strains are more divergent from Me49 than the Ugandan type III is from VEG. The estimated times of MRCA using the intronic mutation rate were in the region of 10^4 ^years, more or less corresponding to the timing of the proposed clonal expansion [17,26]. Interestingly, chromosome VIIa had a higher level of similarity with Me49 than the other type II chromosomes and it may originate from a strain that diverged at a later time point. Although limited data exist for the other Ugandan strains, the slow-growing TgCkUg5 and TgCkUg9 seem to be more closely related to Me49 then the other Ugandan type II strains are. In conclusion, these data point towards a partial separation between the different type II populations starting around 10^4 ^years ago, but genetic exchange may occasionally have occurred between the groups, to give rise to the strains we see today, where different chromosomes have a different level of divergence.

Genome scale analysis of natural recombinant and atypical isolates provide a good tool for mapping genes important for survival and adaptation to different environments. The sequencing of TgCkUg2 generated over 1,200 new SNPs, which were non-randomly distributed in the genome and had a dN/dS ratio greater than 2, suggesting significant selective pressure. Pathogenicity in *T. gondii *is a multigenic trait and a number of loci and genes associated with virulence have been identified, including those encoding rhoptry proteins (ROPs), dense granule proteins (GRAs) and SAG1 related surface antigens (SRSs) [41-45]. In this study, the recombinant type II/III strain caused significantly higher tissue burdens in mice compared with the related type II isolates, but it did not reach the elevated parasite densities of the type III isolate. This intermediate phenotype *in vivo *may be explained by the presence of the virulence-associated alleles for a subset of genes. Indeed, out of the major virulence loci identified by Saeij *et al*. [41], TgCkUg2 possessed the virulent allele for two loci (on chromosomes VIIa and XII) and the avirulent allele for two (on chromosomes VIIb and X). The gene responsible for the quantitative trait locus on VIIa is that encoding ROP18 [41,42], where genotype II is associated with mouse virulence, but no polymorphisms were seen for ROP18 in TgCkUg2 - it was identical to Me49 and the Ugandan type II strains. SAG3 and ROP5 are the candidate genes on XII [41], where genotype III is the virulent type, and while no new SNPs were found for TgCkUg2 in the SAG3 gene, ROP5 was one of the genes with the highest number of novel SNPs. The presence of six non-synonymous and three synonymous new SNPs suggests that ROP5 may be under selection in TgCkUg2. Additional genes identified through statistical dN/dS analysis to be under selective pressure, or found to contain at least two novel coding SNPs in TgCkUg2, included those encoding GRA3 and several ROPs and SRSs (Tables 3 and 4), which may be important for virulence and host-pathogen interactions. Interestingly, one of the genes (551.m00237) found to be under selective pressure by dN/dS analysis is located within the proposed virulence locus on chromosome XII, next to the candidate gene ROP5. While it is currently not possible to determine the exact genes responsible for the intermediate phenotype of this recombinant strain, the high density of SNP data from the current study will provide the basis for closer mapping of genotype-phenotype associations and genes under selection for niche adaptation within African isolates.

## Conclusions

*T. gondii *is an important pathogen with unusual population structure. Although highly zoonotic, it is genetically conserved; thus, considerable depth of study is required to understand genotype phenotype relationships. There are three major outstanding questions about the population genetics of this parasite. Firstly, does recombination occur and, if so, why do clonal genotypes dominate? Secondly, what is the extent of allelic variation among strains? Finally, does allelic variation reflect biogeographical segregation and divergence or are there loci under selection that might dictate the parasite phenotype?

Our study advances understanding of all three of these questions. We find that TgCkUg2, a natural type II/III recombinant strain, has arisen via chromosome sorting and there is no evidence for recent intrachromosomal recombination. Detailed mapping revealed 1,252 unique SNPs within TgCkUg2, which place the divergence of this African strain in relation to the annotated genomes of the North American-derived reference type II and III isolates at around 10^4 ^years ago. Direct sequencing confirms the presence of unique SNPs in additional African isolates, implying that these polymorphisms represent local allelic variants. Estimation of dN/dS ratios across the genome of TgCkUg2 provides evidence of loci under selection. The intermediate phenotype of the recombinant strain provides a reference point for interpretation of the role of candidate virulence genes.

The substantial body of data generated from this study will inform future strategies for analysis of parasite population structure, recombination frequency and gene flow. The existence of the recombinant and closely related parental strains further provide a platform for further genotype-phenotype studies.

## Materials and methods

### Parasite isolation and maintenance

Eight *T. gondii *strains were isolated in mice from Ugandan chickens. These strains are designated as TgCkUg 1, 2, 3, 5, 6, 7, 8 and 9 obtained from chickens 1, 2, 17, 68, 70, 79, 81 and 82, respectively [19]. Strains were retrieved from liquid nitrogen, passaged once in Swiss-Webster mice (at the United States Department of Agriculture (USDA)) and homogenized brain material was thereafter injected into three inbred NMRI mice (at the Swedish Institute for Infectious Disease Control (SMI)) for each isolate. The mice were kept 6 weeks and thereafter put to death humanely. Heart, brain, quadriceps muscle and lung tissue were dissected for quantification of parasite burden, and parasites from the brain were transferred to human foreskin fibroblast cell culture. The brains were homogenized in 1 ml sterile phosphate-buffered saline and subject to digestion in 0.5% trypsin-EDTA for 30 minutes at 37°C, in order to release bradyzoites from their tissue cysts [46]. The parasites were kept in cell culture under standard conditions in Dulbecco's minimum essential medium with 10% foetal bovine serum, 1% HEPES and 1% penicillin and streptomycin. Parasites were regularly passaged to new human foreskin fibroblast cells. All animal work was performed according to national and international guidelines.

### Whole genome sequencing of a natural recombinant strain

Parasites of the TgCkUg2 strain were harvested from cell culture and intracellular parasites were released by needle passage and separated from host cells through centrifugation. DNA was extracted using the QIAamp DNA extraction kit (QIAgen GmbH, Hilden, Germany) and adjusted to a concentration of 100 ng/μl to a total of 5 μg.

We generated 1,480,197 reads in total in three full runs of the 454GSFLX sequencer (Roche, Basel, Switzerland), which resulted in 328,210,041 bases of sequence. In mapping to the Me49 sequence from ToxoDB v4.3, 49.7% of the reads mapped to the genome using the default parameters of the mapping algorithm in the Newbler Software package (Roche); 7.6% of the reads did not map due to being in repetitive regions. The remaining 43% of the sequence contained either host sequence (54.7%) or *T. gondii *sequence that fell below the mapping criteria (27.7%). The remaining sequences are either missing from the reference or due to other contamination within the sample. After mapping we generated 64,721 contigs of 49,866,158 bp in total. This is somewhat shorter than the reference (60,179,518 bp) due to repetitive regions, which cannot be mapped to. The average coverage of the mapped portion of the genome was therefore 4×.

### SNP analysis

The sequence of TgCkUg2 was aligned to the annotated sequence of Me49 (ToxoDB v4.3), and thereafter to VEG, which had first been mapped to the Me49 sequence. SNPs were called when at least three reads, of which at least one was in each direction, called a SNP with 100% concordance. SNPs called in regions that mapped to several places in the genome or SNPs where all reads were not in agreement were dismissed. These strict criteria were used to prevent false SNP calling, but are likely to miss a large proportion of real polymorphisms. To assess this rate, each chromosome was queried for polymorphisms between VEG and Me49 in ToxoDB and compared with the SNPs detected in our analysis, and we found that the SNP detection rate was between 22 and 24%.

The sequence data were analyzed in the sequence viewer and annotation tool Artemis after pre-processing with bespoke Perl scripts, and the annotation for Me49 (ToxoDB v4.3) was used. The three main SNP types are shown in green for (TgCkUg2 = Me49) ≠ VEG, blue for (TgCkUg2 = VEG) ≠ Me49 and orange for TgCkUg2 ≠ (Me49 = VEG). The SNPs were imported from Artemis to Excel for calculations and creation of the SNP density graphs (Additional data file 1). Since TgCkUg2 was a type II/III recombinant, it was not aligned on the whole genome level with the type I reference strain GT1 as this would have greatly increased the complexity of the analysis while adding little useful information. However, in the case of additional targeted sequencing of all Ugandan isolates, comparisons were made with all type I, II and III reference strains. The distribution of SNPs in TgCkUg was also mapped against maps of SNP dominance generated from reference strains as described below.

To compare the polymorphisms in TgCkUg2 to those in the three reference strains (type I, GT-1; type II, ME49; and type III, VEG), all predicted polymorphisms among the type I, II and III strains (kindly provided by Amit Bahl and David Roos, University of Pennsylvania) were used to create SNP type plots as follows. Starting from the left end of each chromosome, a running sum was computed at each SNP position; a type I SNP counted 0, a type II SNP counted +1, and a type III SNP counted -1. This running sum was then plotted against the SNP position on the chromosome (see gray line in Figure 2 and Additional data file 2), and then overlaid to scale on the chromosome maps depicting the degree of similarity between SNPs in TgCkUg2 and type II and type III sequences. On these graphs, therefore, a straight line indicates a chromosomal region where type I SNPs predominate in the canonical type I, II and III strains, a rising line indicates an area of type II dominance, and a falling line indicates an area of type III dominance.

Detection of genes under selection was achieved through dN/dS analysis of all chromosomes using the maximum likelihood method as detailed by Yang [25] and implemented by the codeml program within the Paml (version 4b) software package. Pairwise comparisons of the three strains Me49, VEG and TgCkUg2 were undertaken with each comparison consisting of: a maximum likelihood pairwise comparison to estimate dN/dS (omega); an equivalent maximum likelihood comparison, but with omega fixed at 1; and a likelihood ratio test to assess whether the estimated omega was significantly greater than 1, and thus indicative of positive selection. More detail and a full list of the genes under selection between any of the strains, including the Me49-VEG comparison, is available in Additional data file 3.

### Divergence time estimate

The type II and type III chromosomes of TgCkUg2 were used in parallel to calculate the time of the MRCA of UgII and Me49, and UgIII and VEG, respectively, using two different methodologies. For calculations based on the intron mutation rate for *T. gondii*, the quote of the intron minor SNP count divided by the intron length was divided by the mutation rate 1.94 × 10^-8 ^[26] for all chromosomes separately, and for the entire type II and III regions together. Intronic SNPs were defined as SNPs present in the mRNA, but not in the coding sequence, and the intron lengths were calculated analogously (data were retrieved from Artemis and exported to Excel). The minor SNP type was defined as SNPs against the background (that is, against Me49 on type II chromosomes and against VEG on type III chromosomes), and included novel SNPs.

For calculations based on the type II/III divergence time estimate of 150,000 years [27], the relationship between major and minor SNPs was assumed to be directly proportional to the relationship in time between the ancient and more recent splits. The recombination events between the ancestral strains that gave rise to the present lineages occurred much more recently then did the MRCA of all the present strains. Types II and III have therefore had much less time to diverge in the regions they inherited jointly in this cross - that is, where type I SNPs are predominant [13]. Therefore, this method is not applicable to regions without SNPs between types II and III, and this is why chromosomes Ia and XI and several regions of other chromosomes were excluded. Major SNPs were defined as SNPs against the non-background type (but excluding unique SNPs), and minor SNPs as in the above paragraph.

The *T. gondii *mutation rate on the genome level (including introns, exons and intergenic regions) was calculated, based on the time estimate results from the calculations based on the major-minor SNP relationship, by dividing the quote of the minor SNP count over the sequence length with the new time estimates. Raw data and calculations are provided in Additional data file 4.

### Targeted sequencing

Sanger sequencing confirmed SNPs discovered by the 454 sequencing of TgCkUg2, and was used to investigate the genetic relationship of the Ugandan isolates and reference strains. PCR products were purified using a PCR purification kit (QIAgen) and sequenced using Big Dye Terminator on an ABIPrism sequencer. Reference sequences for GT1, Me49 and VEG were retrieved from ToxoDB. TgCkUg2, 5, 6 and 8 were sequenced for all loci, and the four remaining Ugandan type II strains were sequenced for selected loci where informative polymorphisms were found. Thirty-four different loci were investigated and the primers are listed in Additional data file 5.

### Data deposition

All contigs assembled from the 454 sequencing of TgCkUg2 have been submitted to the Toxoplasma database [14], together with information on all identified SNPs between TgCkUg2 and Me49 or VEG. Incorporation of these data is in progress. The sequence data generated through conventional sequencing from the eight Ugandan strains are available in GenBank, accession numbers [GenBank:FI274744] to [GenBank:FI274973].

### Phenotype *in vivo *and *in vitro*

The parasite density in different mouse organs was determined in mice used for isolation purposes. Each isolate was retrieved from storage at the USDA and inoculated into one mouse, from which half the brain was shipped to the SMI. For each isolate, half a brain was homogenized in phosphate-buffered saline and inoculated into three mice, from which brain, heart, lung and quadriceps muscle were dissected, weighed and used for DNA-extraction. Q-PCR with primers 5'-GCTCCTCCAGCCGTCTTG-3' (forward), 5'-TCCTCACCCTCGCCTTCAT-3' (reverse), together with a 6-FAM labeled probe 5'-AGGAGAGATATCAGGACTGTA-3' (designed by Daniel Palm, SMI) to target the approximately 300-fold repetitive region AF146527 [47]. ROX was used as a passive reference and the program used was 2 minutes at 50°C, 10 minutes at 95°C followed by 45 cycles of 15 s at 95°C and 1 minute at 60°C. All samples were analyzed in duplicate and, in addition, two negative controls and a triplicate tenfold dilution series used to generate the standard curve, ranging from 0.1 to 1,000 parasites/μl of DNA template, were included on every plate.

*In vitro *growth data were collected during the isolates' eighth passage in cell culture. The well-characterized laboratory strain Me49 was included as a reference and studied in parallel with the new isolates. All parasites were grown in T25 flasks in exactly 6 ml of medium and, for each time point, 5 ml were taken for DNA extraction and replaced with fresh medium; thus, only extracellular parasites were quantified. Growth was also continuously monitored in all passages by visual inspection and scoring of intracellular growth.

## Abbreviations

dN/dS: non-synonymous to synonymous mutation rate; GRA: dense granule protein; MRCA: most recent common ancestor; Q-PCR: quantitative PCR; ROP: rhoptry protein; SMI: Swedish Institute for Infectious Disease Control; SNP: single nucleotide polymorphism; SRS: SAG 1 like surface antigen; USDA: United States Department of Agriculture.

## Authors' contributions

ILB carried all studies on isolation, phenotyping and sequencing of *Toxoplasma *strains; advice and support was provided by JPD for *Toxoplasma *strain isolation and by JL for phenotyping studies. NH designed and implemented the 454 sequencing strategy and, together with KEA, assimilated genome data into contigs. NH, KEA and ILB undertook SNP mapping and analysis, ILB completed MRCA analysis, KEA completed the dN/dS screen and JPB mapped data against SNP profiles from ToxoDB. JES and ILB conceived the study, and participated in its design and coordination. ILB and JES drafted the manuscript. All authors provided comments on the paper and read and approved the final manuscript.

## Additional data files

The following additional data are available with the online version of this paper: graphs showing the distribution of SNPs called from the 454 whole genome sequencing of the recombinant Ugandan *T. gondii *strain TgCkUg2 (Additional data file 1); overlay pictures for all 14 chromosomes that compare the distribution of SNPs in TgCkUg2 to the dominant SNP types for the three *Toxoplasma *reference strains (Additional data file 2); a table showing maximum likelihood analysis of SNPs, listing loci where omega (dN/dS) is significantly greater than 1 (Additional data file 3); a table showing original data and calculations for divergence time, using either the intron mutation rate of 1.94 × 10^-8 ^or the MRCA estimate of 150,000 years for calibration of the molecular clock (Additional data file 4); a table of primers used for sequence-based genotyping of *Toxoplasma *isolates (Additional data file 5).

## Supplementary Material

Additional data file 1 A PDF file showing distribution of SNPs called from the 454 whole genome sequencing of the recombinant Ugandan *T. gondii *strain TgCkUg2. Click here for file

Additional data file 2 A PDF file showing comparison of the distribution of SNPs in TgCkUg2 to the dominant SNP types for the three *Toxoplasma *reference strains for all 14 chromosomes. Click here for file

Additional data file 3 A PDF file showing maximum likelihood analysis of SNPs, listing loci where omega (dN/dS) is significantly greater than 1. Click here for file

Additional data file 4 An Excel file showing original data and calculations for divergence time, using either the intron mutation rate of 1.94 × 10^-8 ^or the MRCA estimate of 150,000 years for calibration of the molecular clock. Click here for file

Additional data file 5 An Excel file showing primers used for sequence-based genotyping of *Toxoplasma *isolates. Click here for file
