# Supplementary material for: Whole genome sequencing of a natural recombinant Toxoplasma gondii strain reveals chromosome sorting and local allelic variants
Source: Genome Biol. 2009 May 20;10(5):R53. doi: 10.1186/gb-2009-10-5-r53 (PMC2718519; doi:10.1186/gb-2009-10-5-r53)
Supplement: Additional data file 1 — A PDF file showing distribution of SNPs called from the 454 whole genome sequencing of the recombinant Ugandan T. gondii strain TgCkUg2. [file gb-2009-10-5-r53-S1.pdf]

Additional data file 1.

Graphs showing the distribution of SNPs called from the 454 whole genome sequencing of the recombinant Ugandan *Toxoplasma gondii* strain TgCkUg2 in the comparison with Me49 (type II), VEG (type III) or both.
Green colour indicates SNPs where TgCkUg2 is identical to Me49 (type II background), blue indicates SNPs where TgCkUg2 is identical to VEG (type III background), and orange SNPs represent novel polymorphisms, where TgCkUg2 had a different allele from both Me49 and VEG. The number of SNPs of each kind is shown for all chromosomes in intervals of 10 kb.

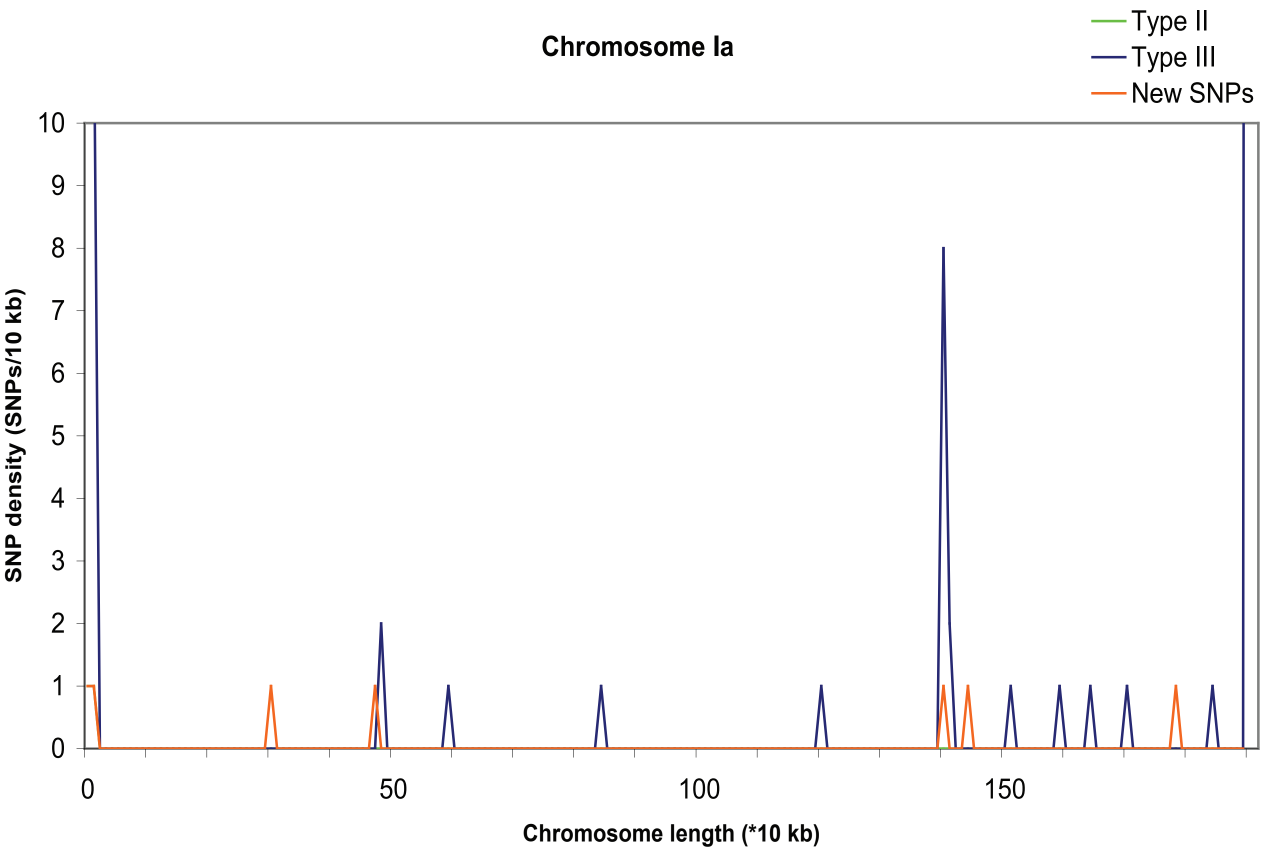
Chromosome Ia
Type II
Type III
New SNPs
SNP density (SNPs/10 kb)
0
1
2
3
4
5
6
7
8
9
10
0
50
100
150
Chromosome length (*10 kb)

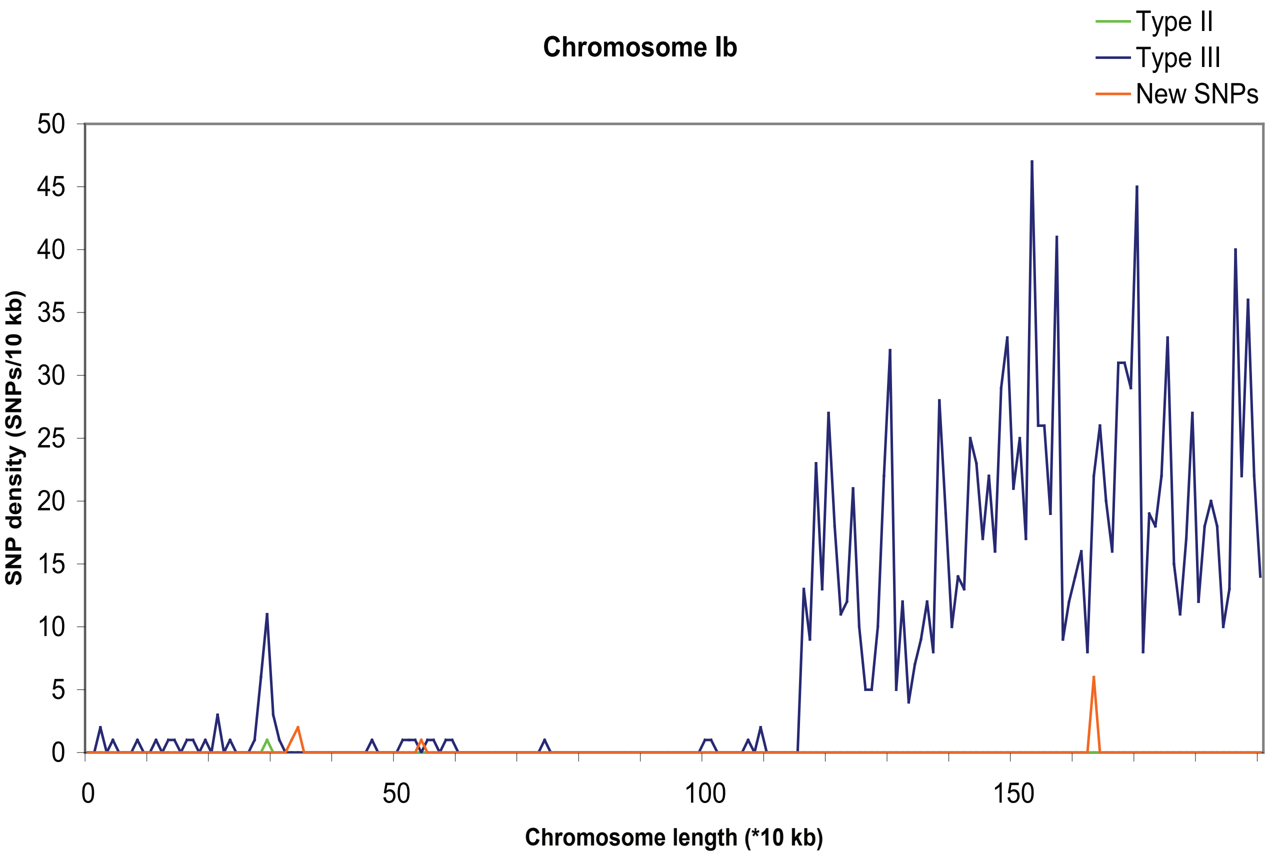

Chromosome Ib
Type II
Type III
New SNPs
SNP density (SNPs/10 kb)
0
5
10
15
20
25
30
35
40
45
50
0
50
100
150
Chromosome length (*10 kb)

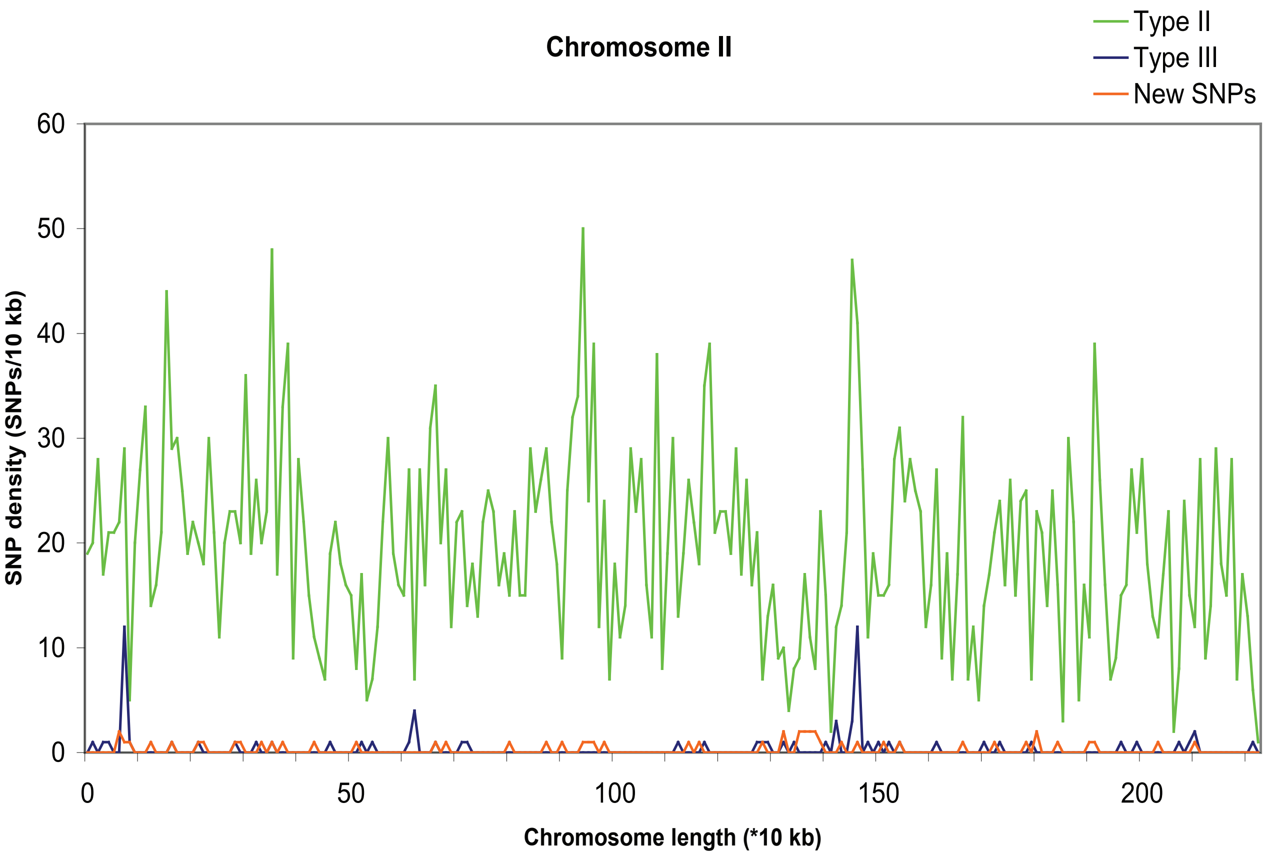

Chromosome II
Type II
Type III
New SNPs
SNP density (SNPs/10 kb)
60
50
40
30
20
10
0
0
50
100
150
200
Chromosome length (*10 kb)

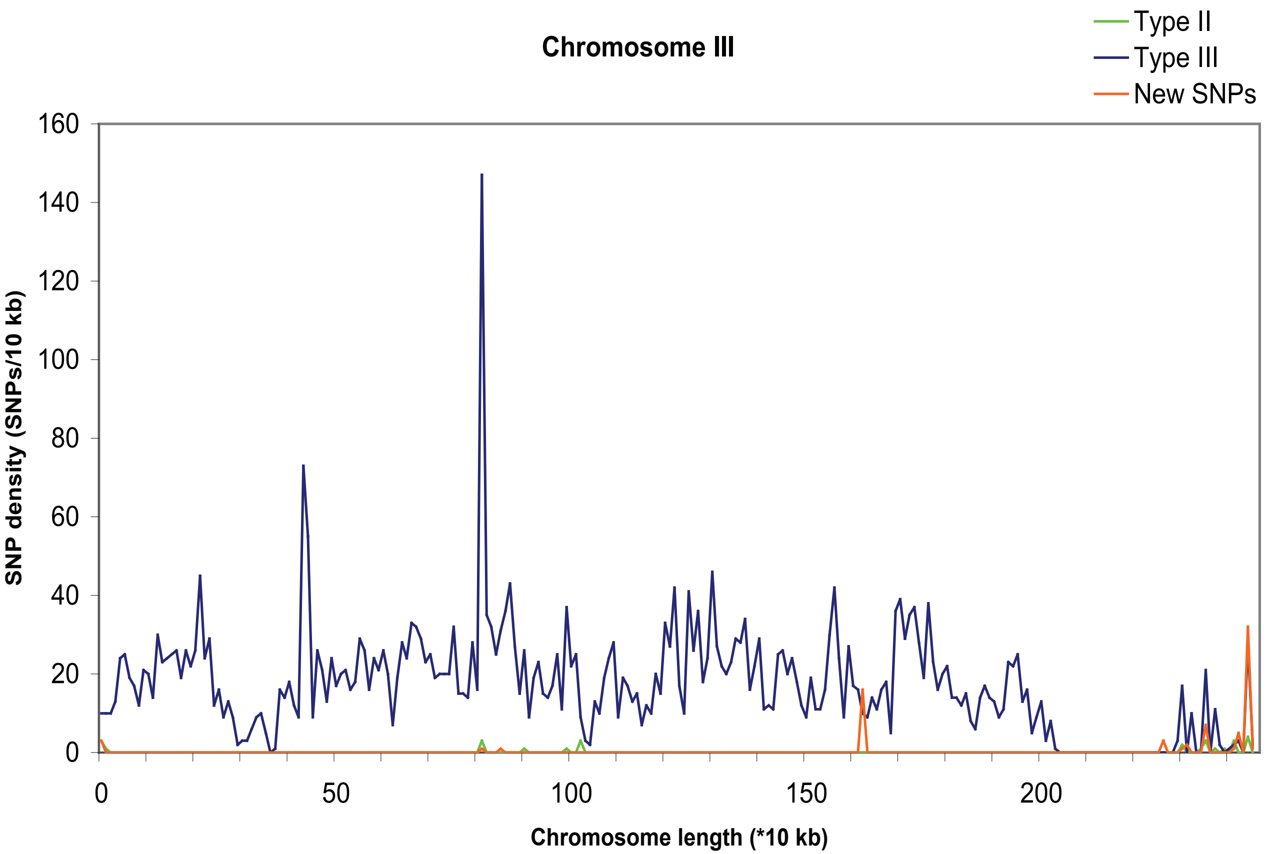
Chromosome III
Type II
Type III
New SNPs
SNP density (SNPs/10 kb)
160
140
120
100
80
60
40
20
0
0
50
100
150
200
Chromosome length (*10 kb)

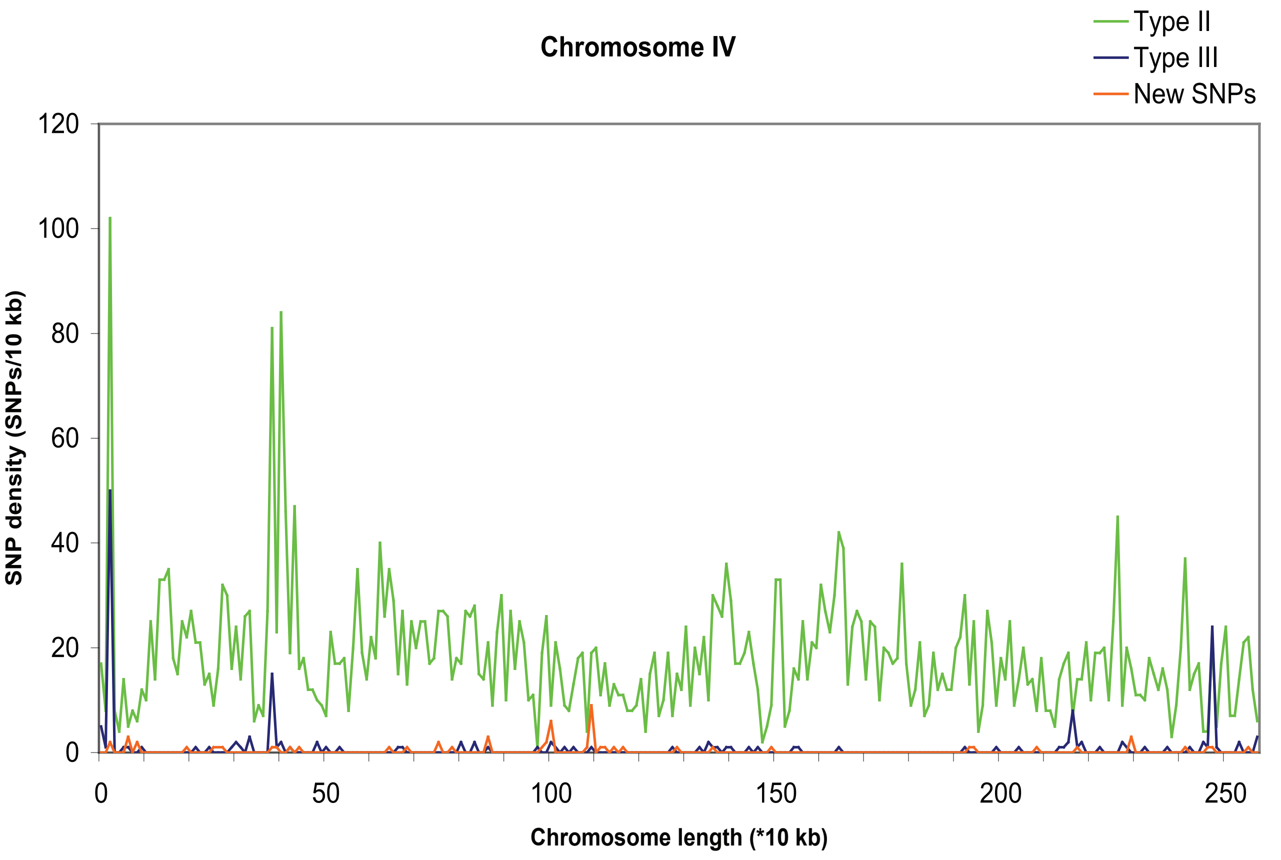
Chromosome IV
Type II
Type III
New SNPs
SNP density (SNPs/10 kb)
0
20
40
60
80
100
120
0
50
100
150
200
250
Chromosome length (*10 kb)

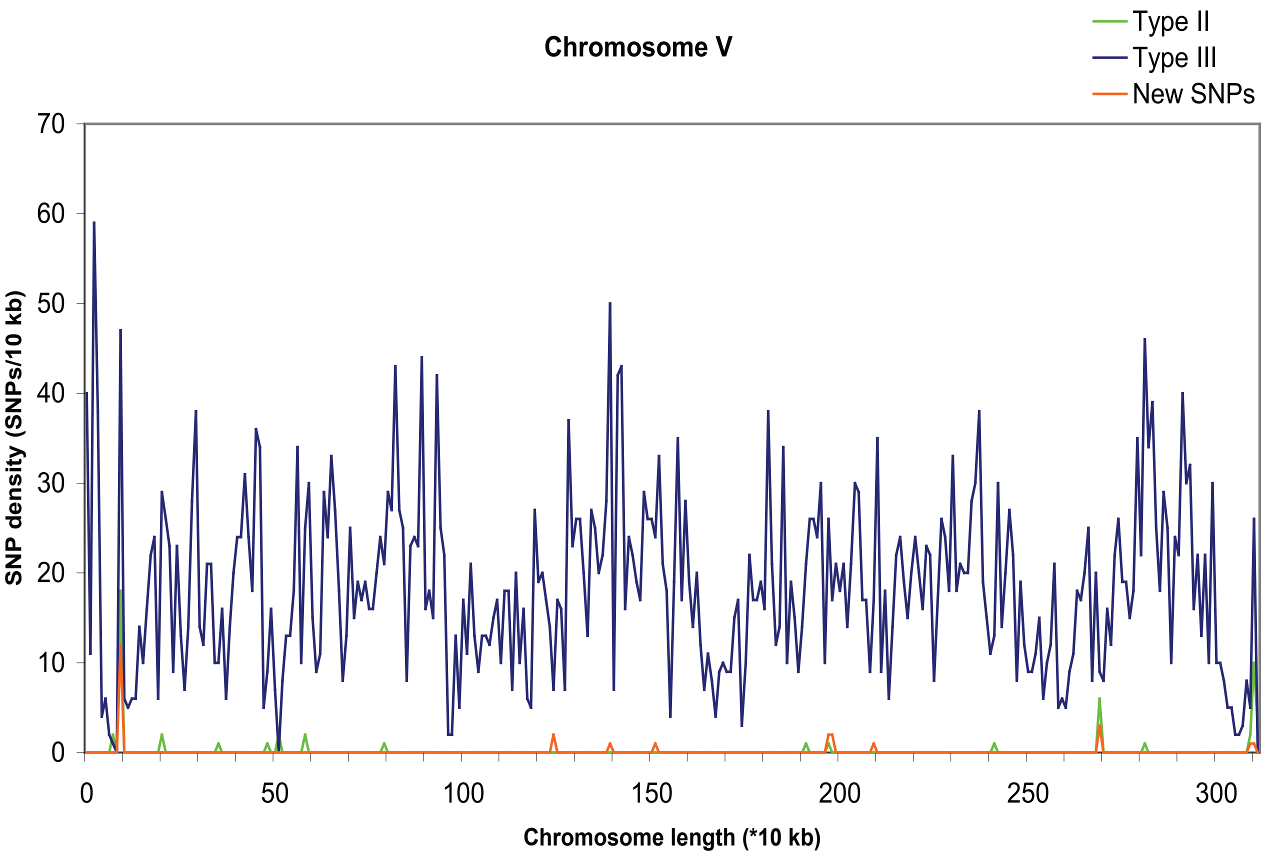
Chromosome V
Type II
Type III
New SNPs
SNP density (SNPs/10 kb)
Chromosome length (*10 kb)
0
10
20
30
40
50
60
70
0
50
100
150
200
250
300

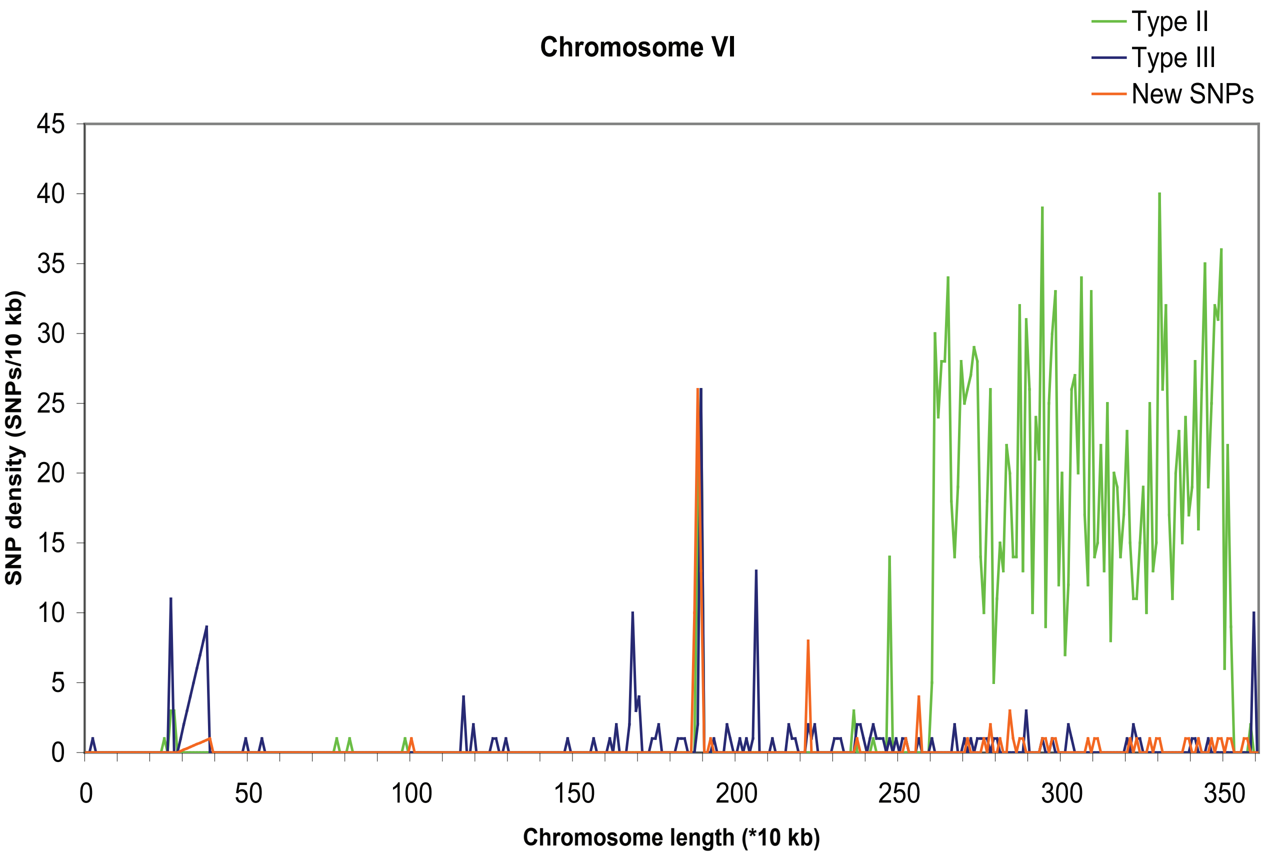
Chromosome VI
Type II
Type III
New SNPs
SNP density (SNPs/10 kb)
0
5
10
15
20
25
30
35
40
45
0
50
100
150
200
250
300
350
Chromosome length (*10 kb)

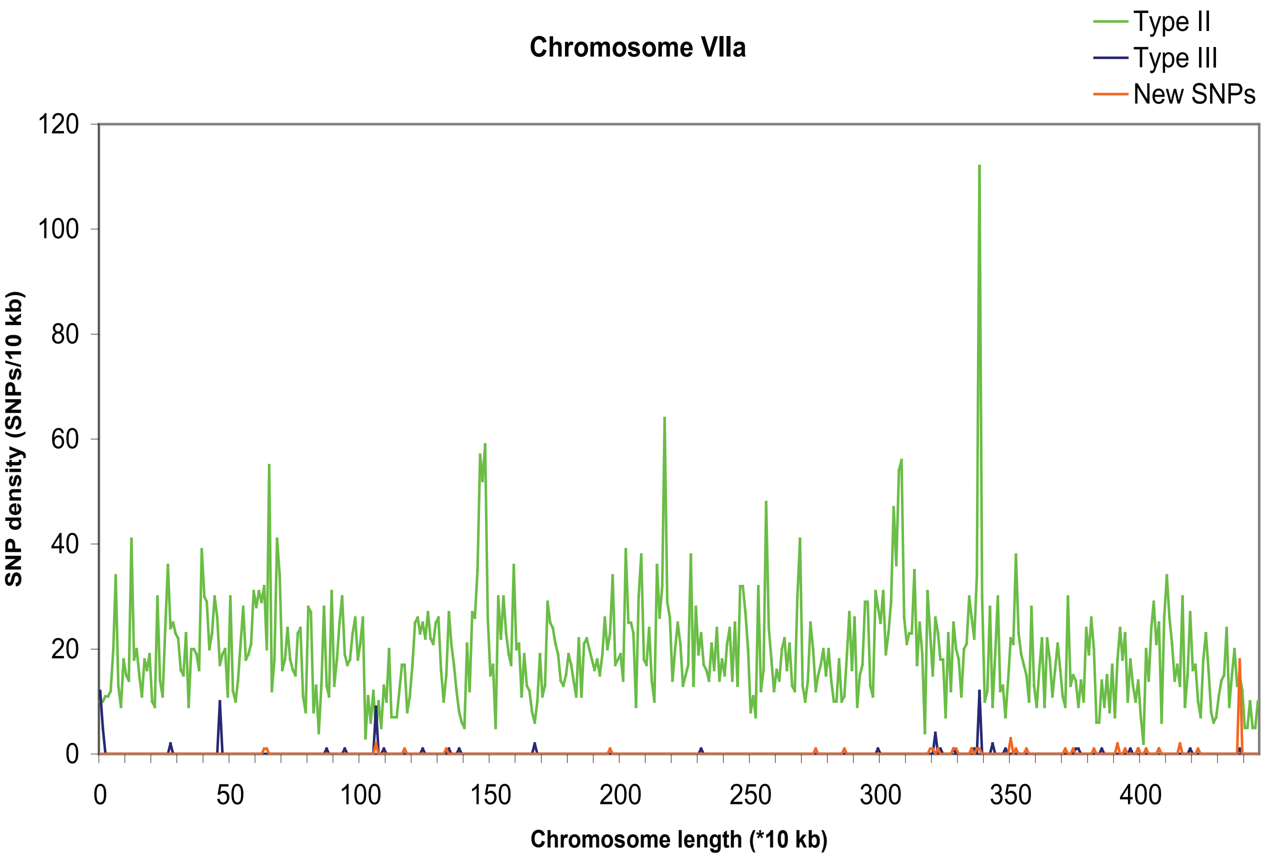

Chromosome VIIa
Type II
Type III
New SNPs
SNP density (SNPs/10 kb)
0
20
40
60
80
100
120
0
50
100
150
200
250
300
350
400
Chromosome length (*10 kb)

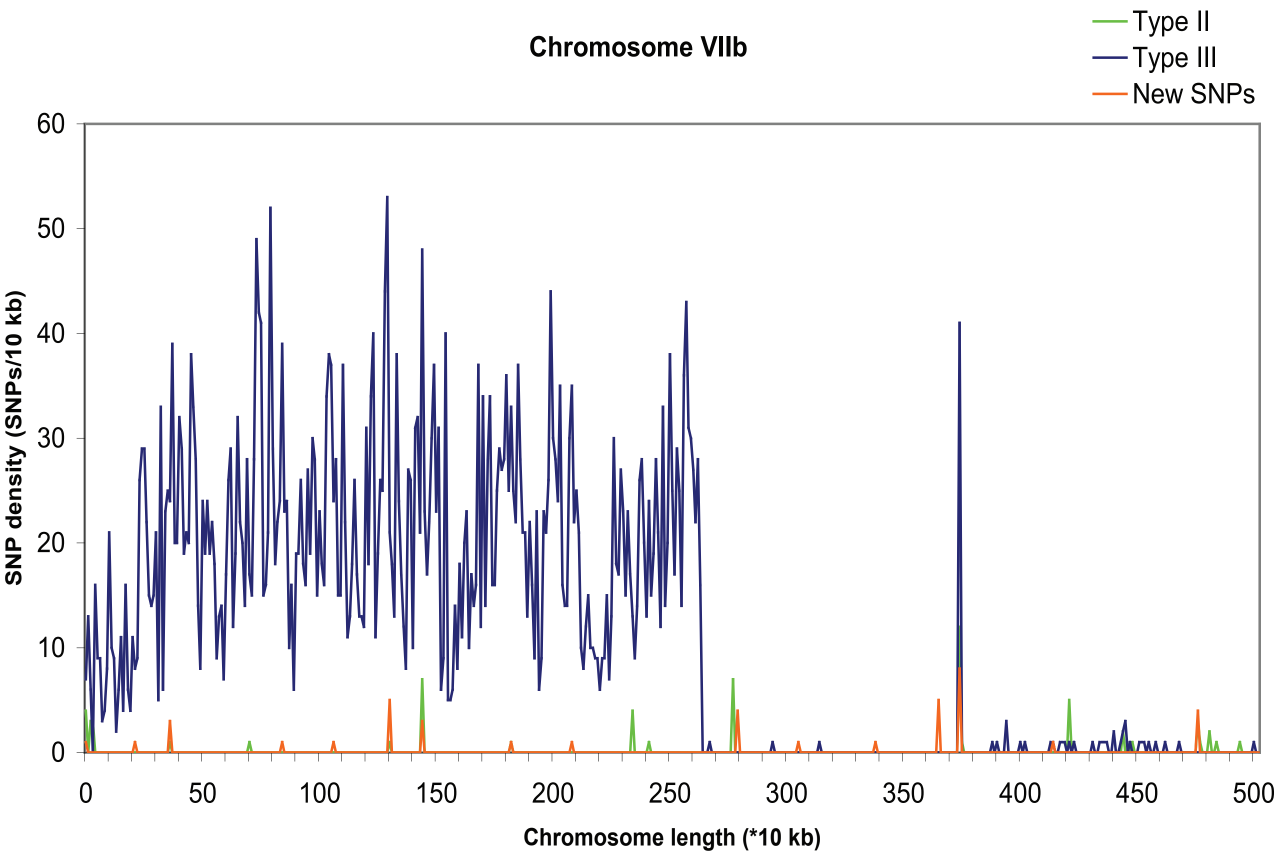
Chromosome VIIb
Type II
Type III
New SNPs
SNP density (SNPs/10 kb)
60
50
40
30
20
10
0
0
50
100
150
200
250
300
350
400
450
500
Chromosome length (*10 kb)

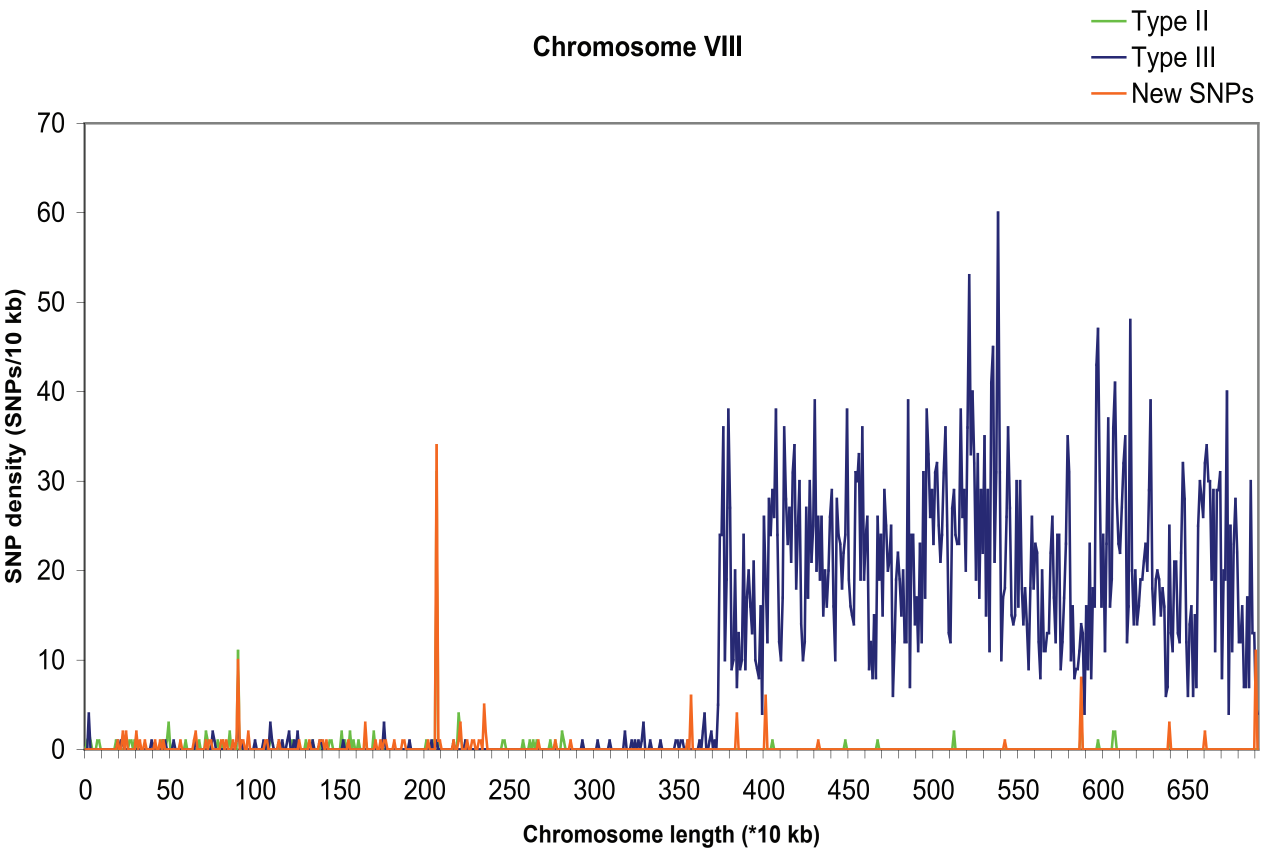
Chromosome VIII
Type II
Type III
New SNPs
SNP density (SNPs/10 kb)
Chromosome length (*10 kb)
0
10
20
30
40
50
60
70
0
50
100
150
200
250
300
350
400
450
500
550
600
650

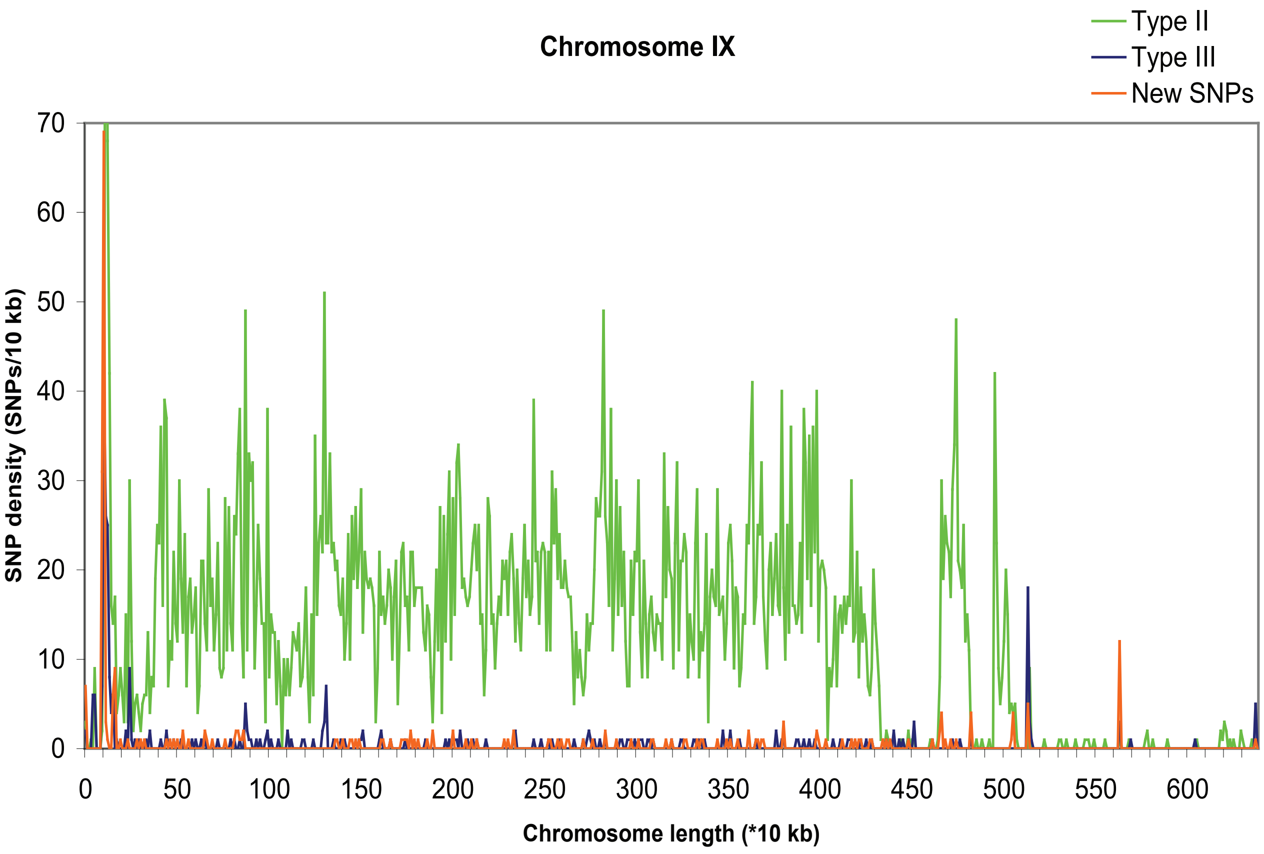
Chromosome IX
Type II
Type III
New SNPs
SNP density (SNPs/10 kb)
0
10
20
30
40
50
60
70
0
50
100
150
200
250
300
350
400
450
500
550
600
Chromosome length (*10 kb)

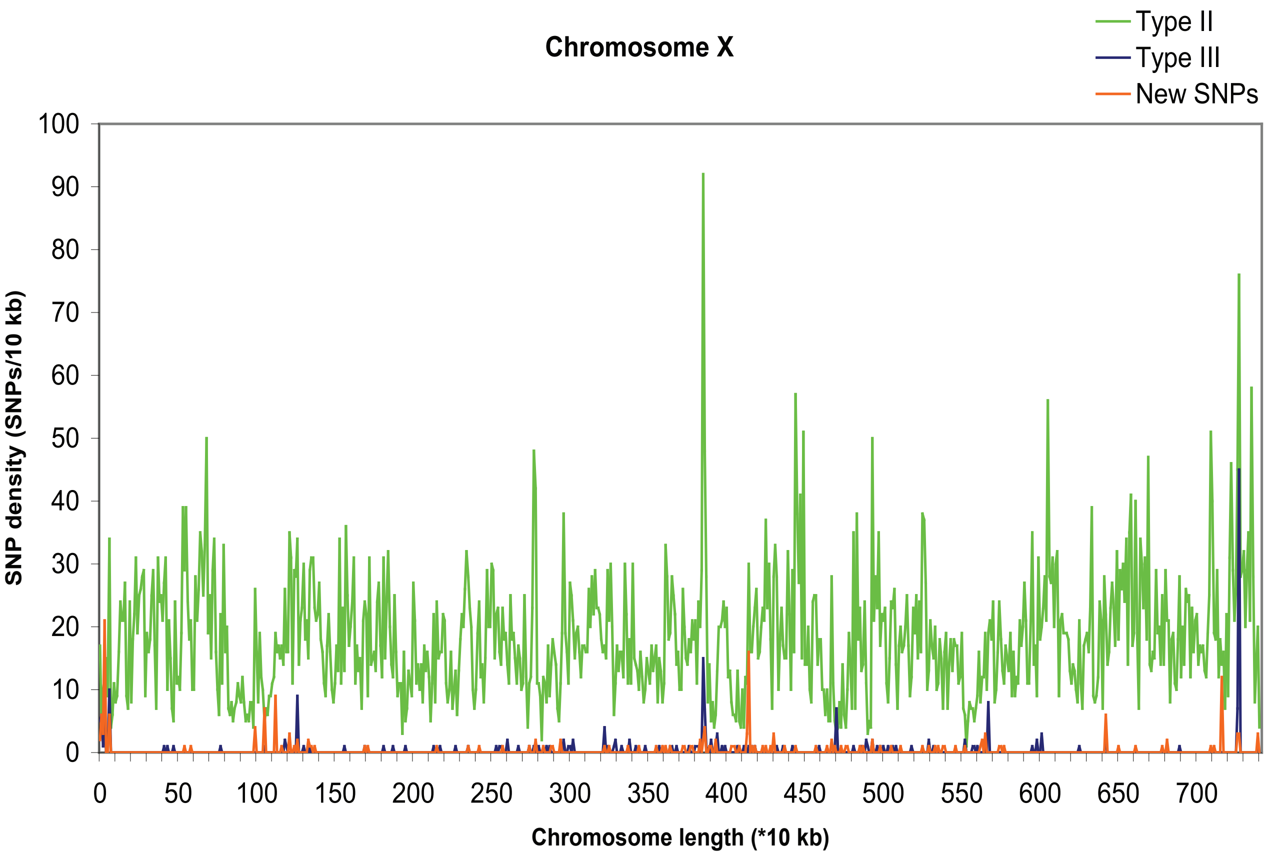

Chromosome X
Type II
Type III
New SNPs
SNP density (SNPs/10 kb)
100
90
80
70
60
50
40
30
20
10
0
0
50
100
150
200
250
300
350
400
450
500
550
600
650
700
Chromosome length (*10 kb)

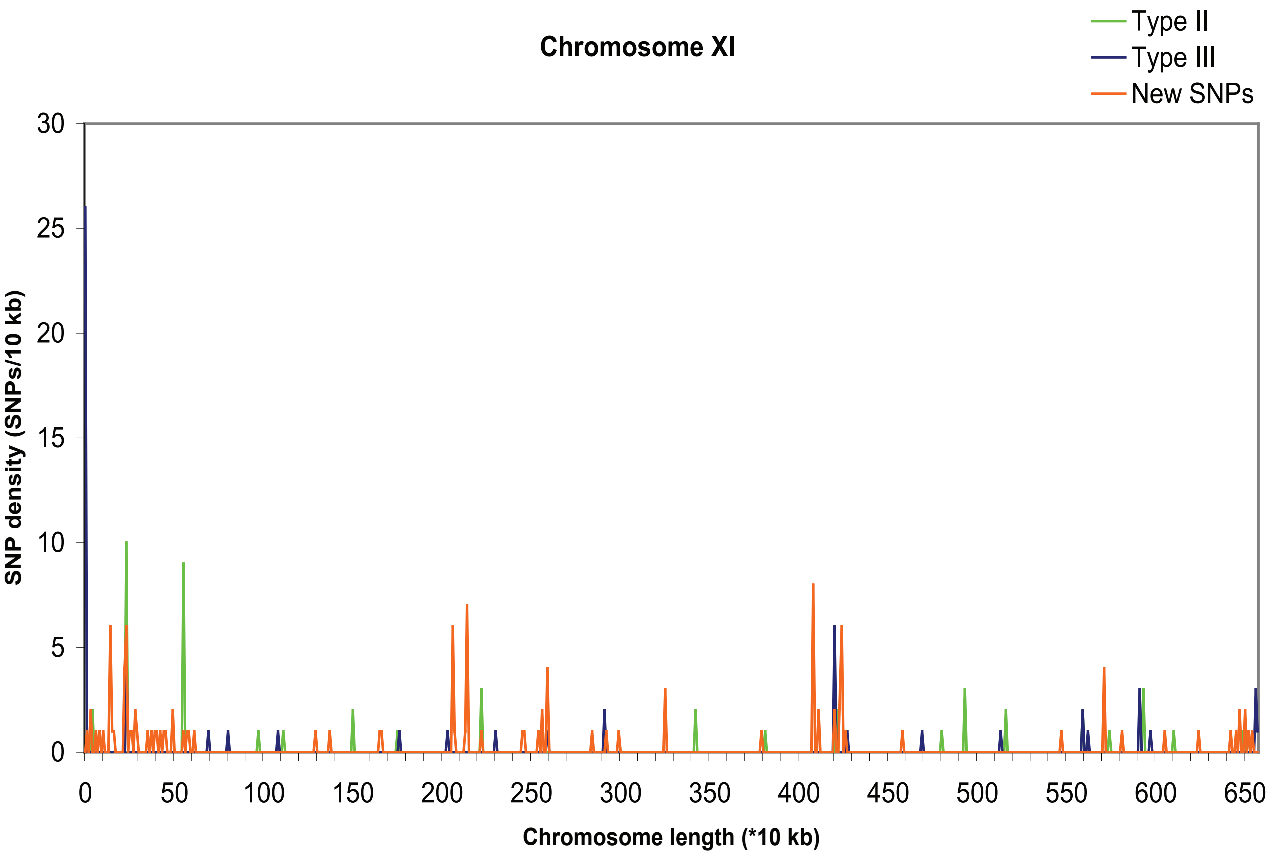

Chromosome XI
Type II
Type III
New SNPs
SNP density (SNPs/10 kb)
30
25
20
15
10
5
0
0
50
100
150
200
250
300
350
400
450
500
550
600
650
Chromosome length (*10 kb)

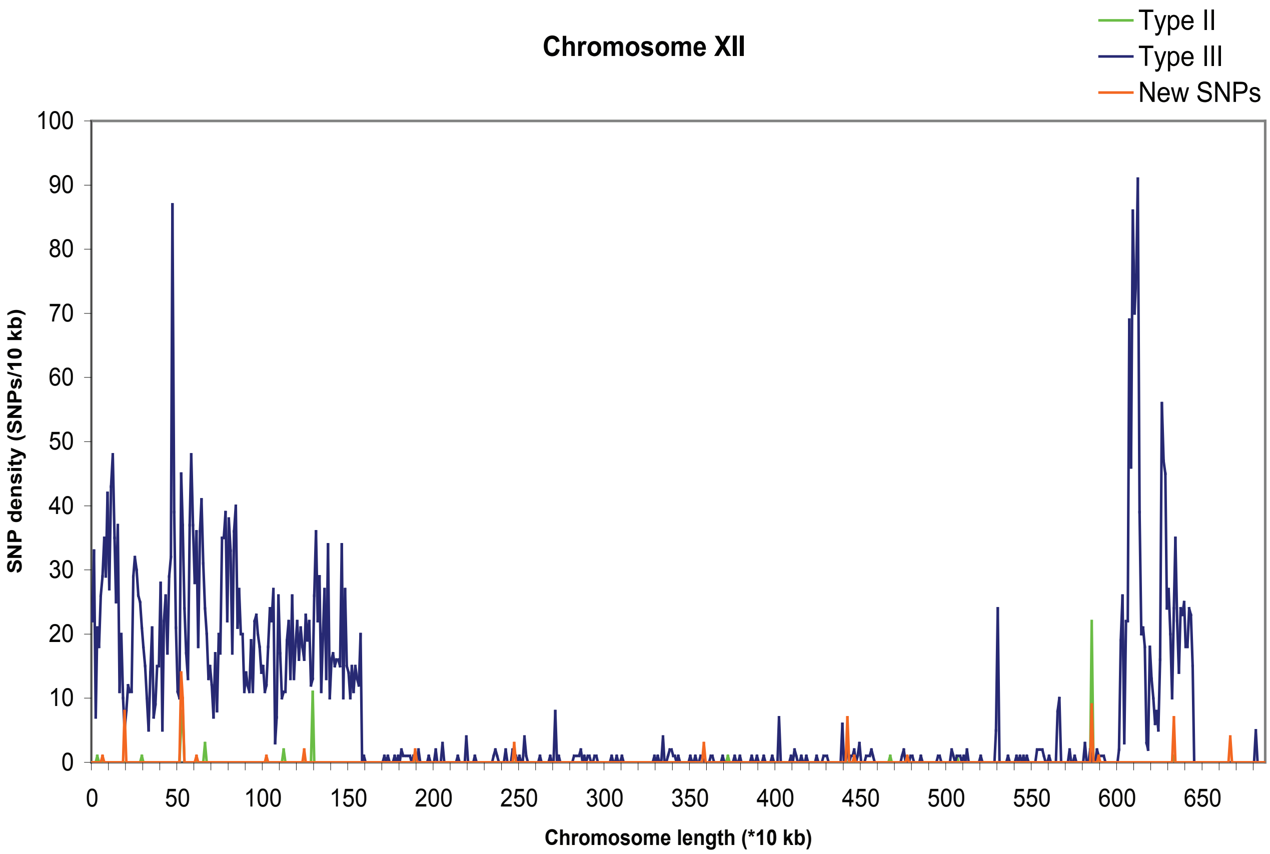

Chromosome XII
Type II
Type III
New SNPs
SNP density (SNPs/10 kb)
100
90
80
70
60
50
40
30
20
10
0
0
50
100
150
200
250
300
350
400
450
500
550
600
650
Chromosome length (*10 kb)
