# Supplementary material for: Whole genome sequencing of a natural recombinant Toxoplasma gondii strain reveals chromosome sorting and local allelic variants
Source: Genome Biol. 2009 May 20;10(5):R53. doi: 10.1186/gb-2009-10-5-r53 (PMC2718519; doi:10.1186/gb-2009-10-5-r53)
Supplement: Additional data file 2 — A PDF file showing comparison of the distribution of SNPs in TgCkUg2 to the dominant SNP types for the three Toxoplasma reference strains for all 14 chromosomes. [file gb-2009-10-5-r53-S2.pdf]

Additional data file 2

Overlay pictures for all 14 chromosomes comparing the SNP patterns in the 3 reference *Toxoplasma* strains to those in TgCkUg2. Graphs were constructed as described in Figure 2. Y-axis scales are not shown but range from 1500 for Chr. Ia and Ib to 16000 for Chr. X. For Chr. VIIa, in the reference strains type III SNPs dominate for the first 0.5 Mb, which is illustrated by the descending grey line. However, since TgCkUg2 acquired this entire chromosome from the type II parent, TgCkUg2 has the type II allele at all polymorphic sites in this region (indicated by the green lines).
In regions where type I SNPs dominate in the reference strains, types II and III are highly similar, and the lack of major SNPs in TgCkUg2 corresponds to the regions where the grey line is flat. This correlation is obvious for the larger regions in Ib, VI, VIIb, VIII, XI and XII, but can also be seen in more detail for several short regions, for example on chromosome IX (in the very beginning and around 4.5 Mb and 4.9 Mb on the X-axis). This close match between these two sets of data strengthens the notion of TgCkUg2 being the progeny of two strains which are highly similar to the archetypal type II and III.

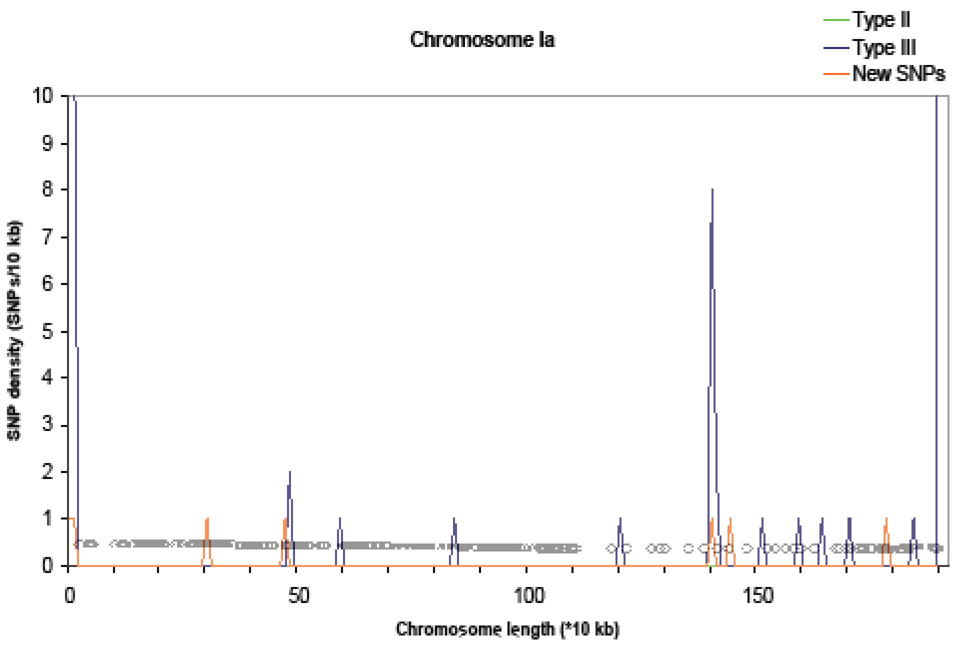

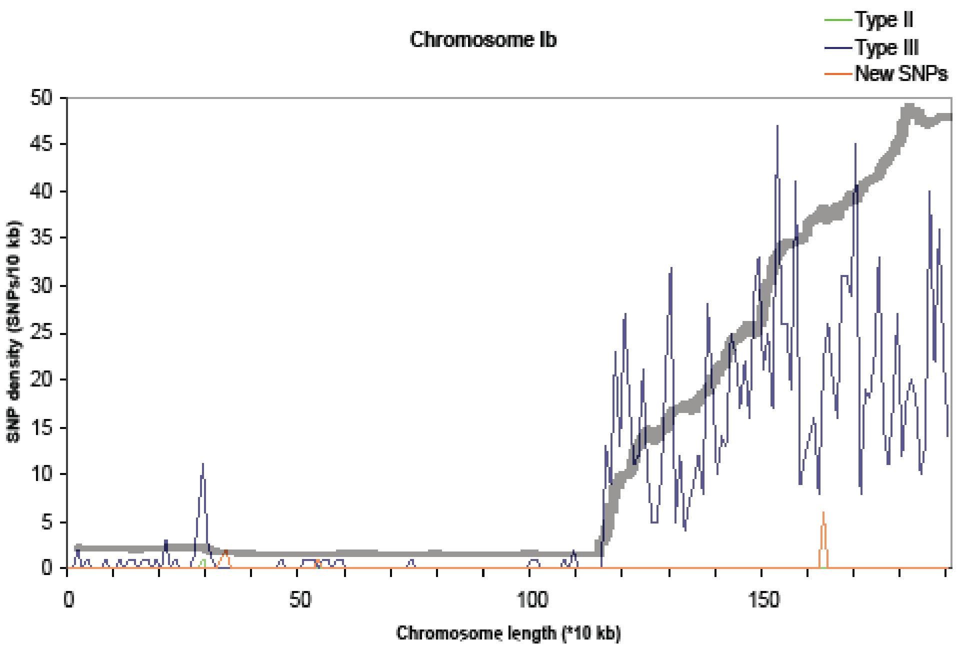

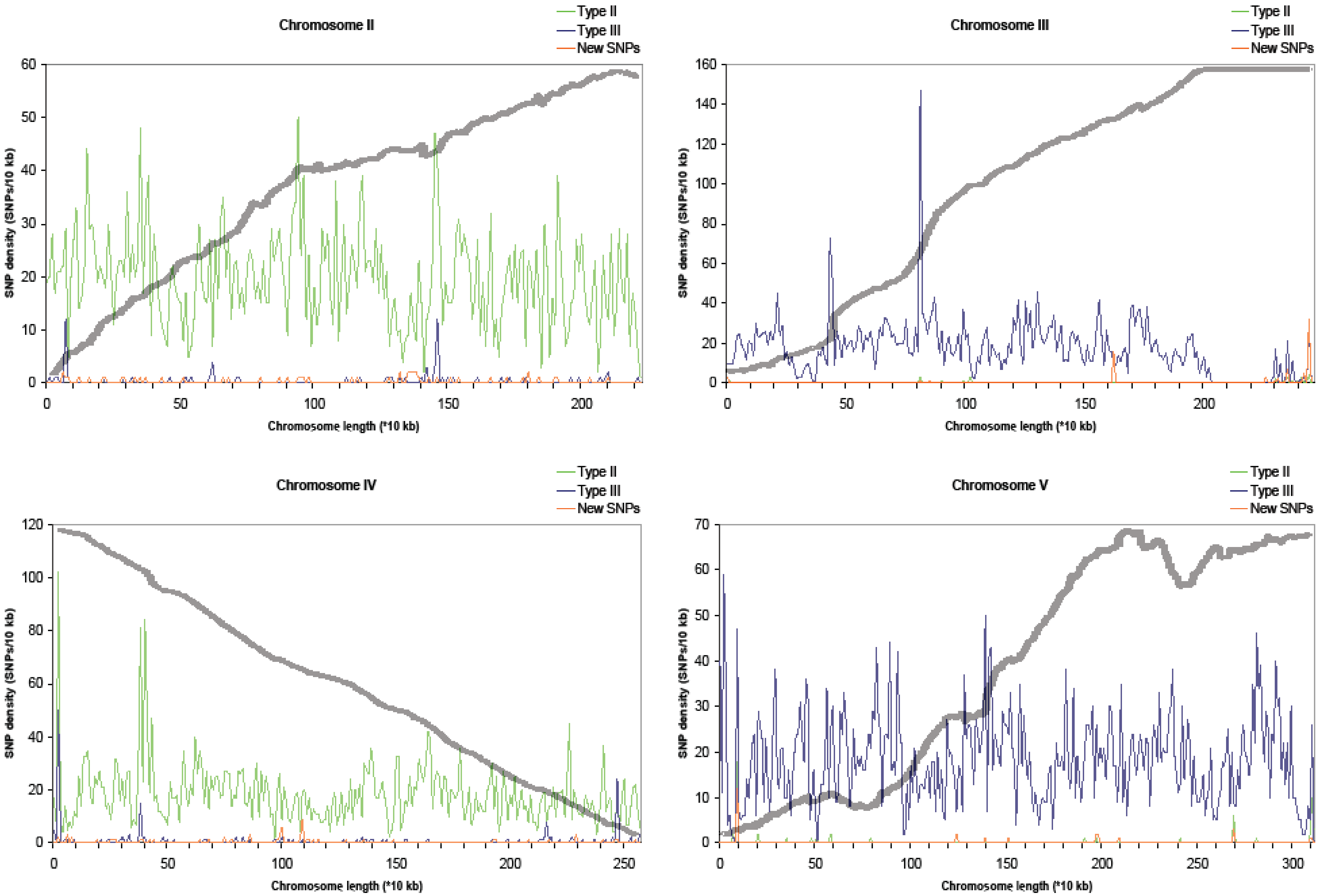

Chromosome II
Type II
Type III
New SNPs
SNP density (SNPs/10 kb)
Chromosome length (*10 kb)
0
10
20
30
40
50
60
0
50
100
150
200
Chromosome III
Type II
Type III
New SNPs
SNP density (SNPs/10 kb)
Chromosome length (*10 kb)
0
20
40
60
80
100
120
140
160
0
50
100
150
200
Chromosome IV
Type II
Type III
New SNPs
SNP density (SNPs/10 kb)
Chromosome length (*10 kb)
0
20
40
60
80
100
120
0
50
100
150
200
250
Chromosome V
Type II
Type III
New SNPs
SNP density (SNPs/10 kb)
Chromosome length (*10 kb)
0
10
20
30
40
50
60
70
0
50
100
150
200
250
300

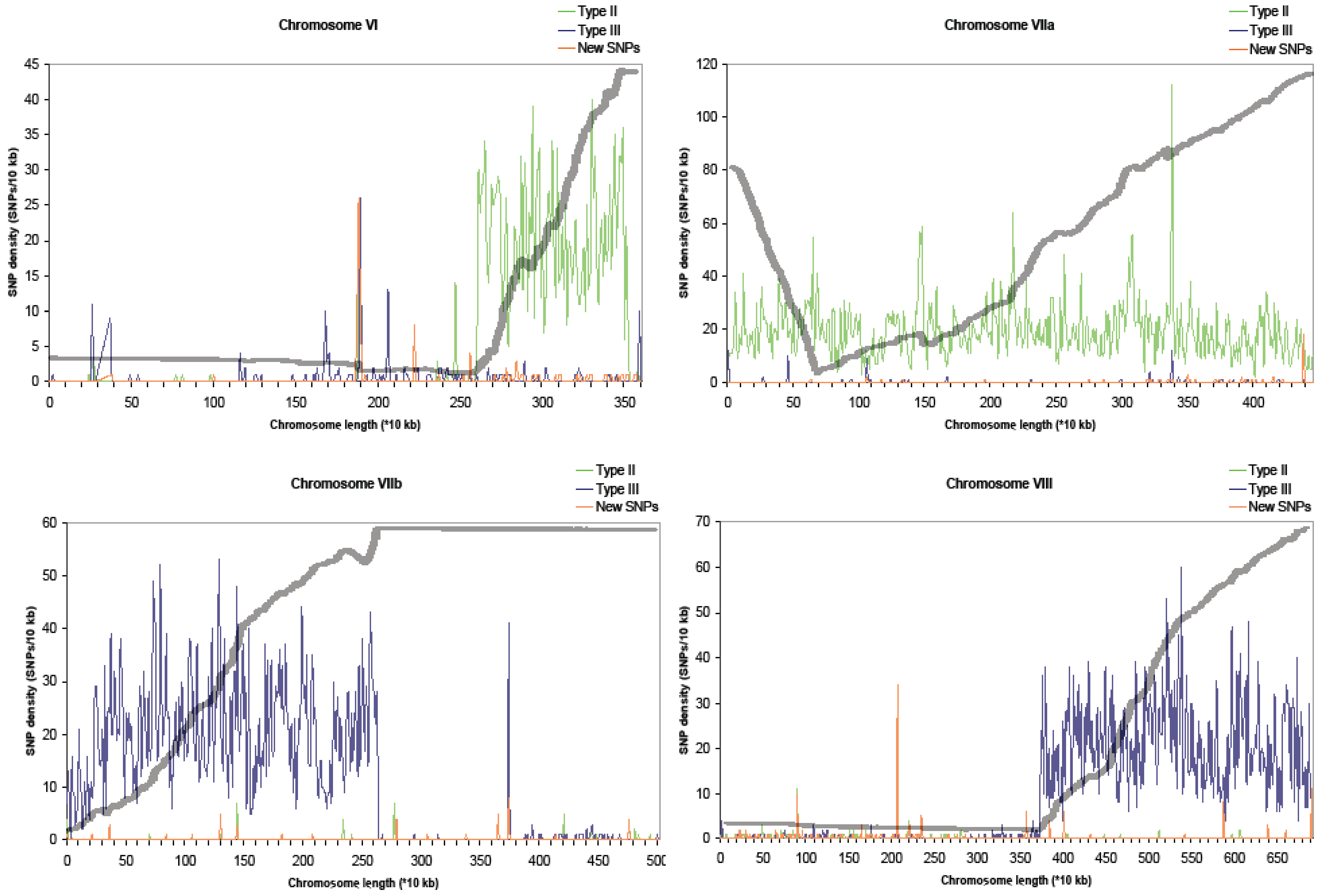
Chromosome VI
Type II
Type III
New SNPs
SNP density (SNPs/10 kb)
Chromosome length (*10 kb)
Chromosome VIIa
Type II
Type III
New SNPs
SNP density (SNPs/10 kb)
Chromosome length (*10 kb)
Chromosome VIIb
Type II
Type III
New SNPs
SNP density (SNPs/10 kb)
Chromosome length (*10 kb)
Chromosome VIII
Type II
Type III
New SNPs
SNP density (SNPs/10 kb)
Chromosome length (*10 kb)

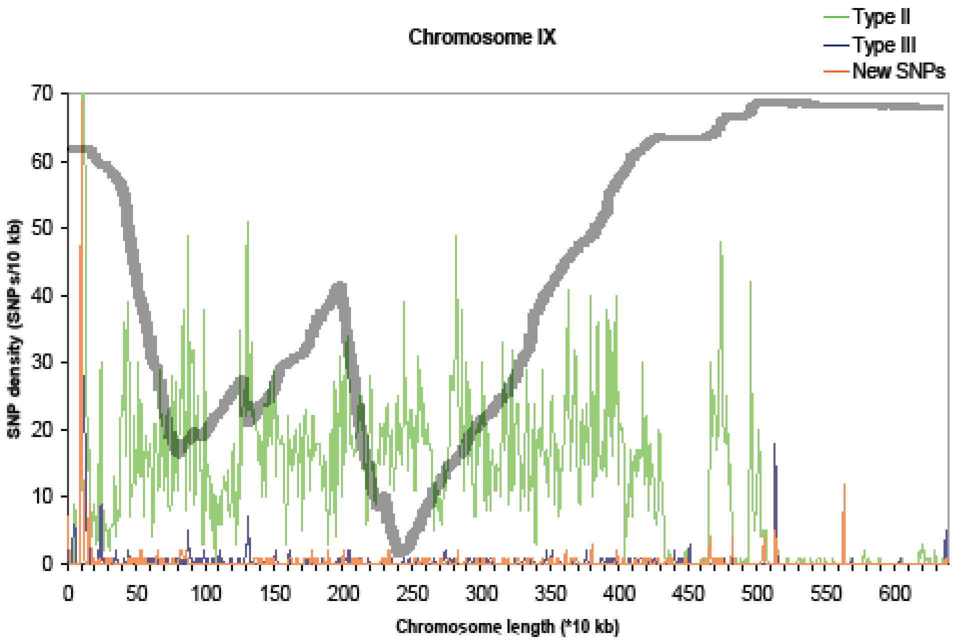
Chromosome IX
Type II
Type III
New SNPs
SNP density (SNPs/10 kb)
Chromosome length (*10 kb)
0
10
20
30
40
50
60
70
0
50
100
150
200
250
300
350
400
450
500
550
600

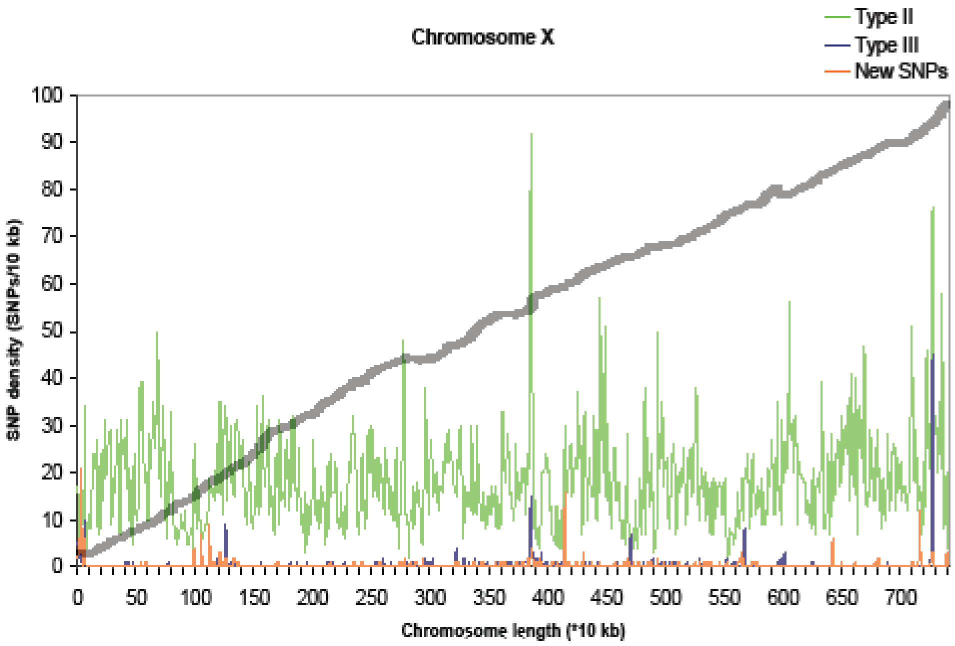
Chromosome X
Type II
Type III
New SNPs
SNP density (SNPs/10 kb)
Chromosome length (*10 kb)
0
10
20
30
40
50
60
70
80
90
100
0
50
100
150
200
250
300
350
400
450
500
550
600
650
700

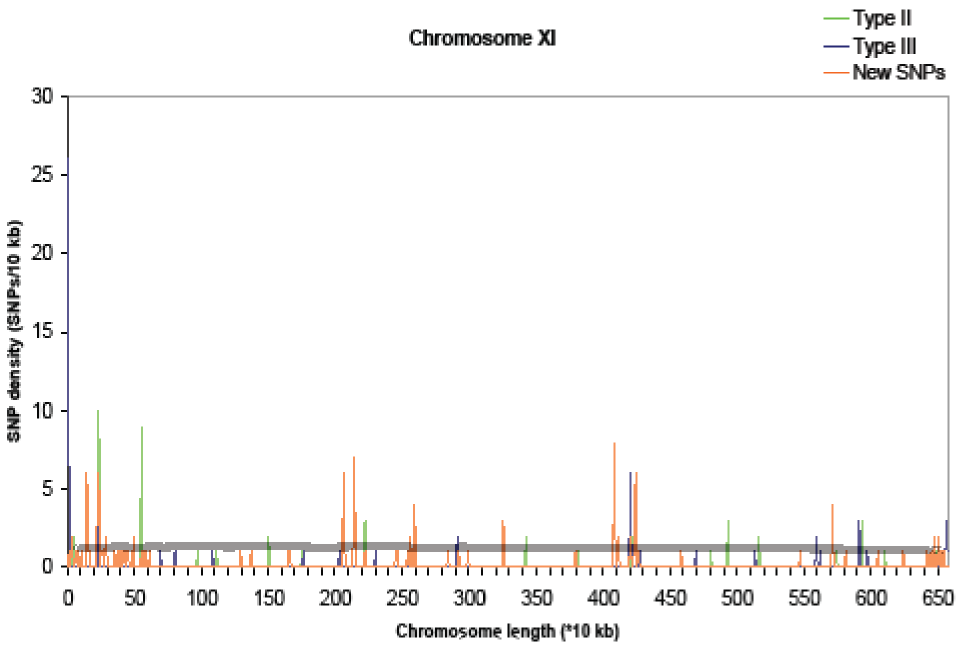
Chromosome XI
Type II
Type III
New SNPs
SNP density (SNPs/10 kb)
Chromosome length (*10 kb)
0
5
10
15
20
25
30
0
50
100
150
200
250
300
350
400
450
500
550
600
650

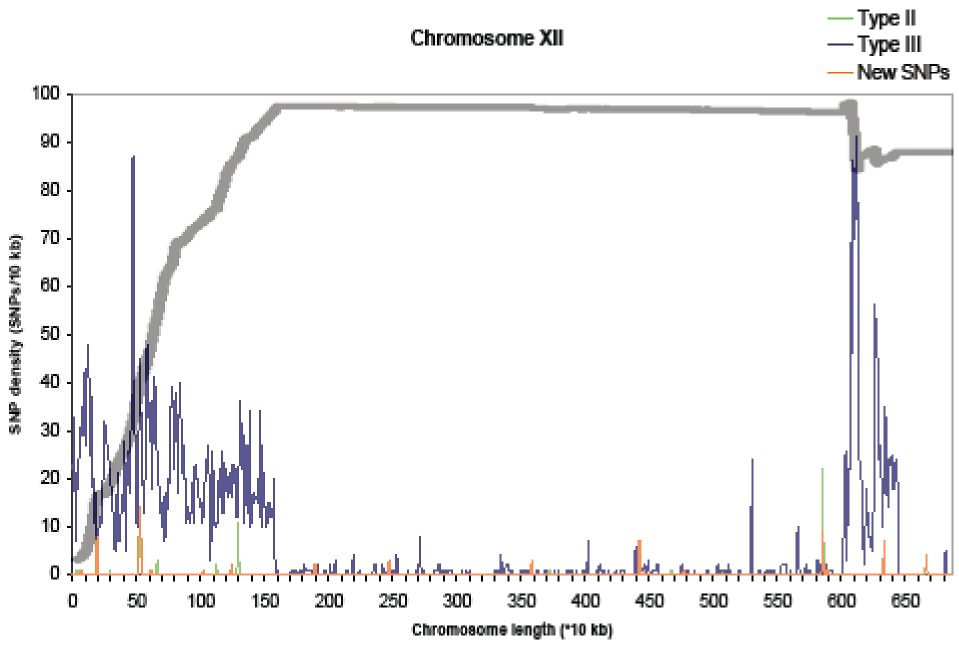
Chromosome XII
Type II
Type III
New SNPs
SNP density (SNPs/10 kb)
Chromosome length (*10 kb)
0
10
20
30
40
50
60
70
80
90
100
0
50
100
150
200
250
300
350
400
450
500
550
600
650
