# Supplementary material for: Whole genome sequencing of a natural recombinant Toxoplasma gondii strain reveals chromosome sorting and local allelic variants
Source: Genome Biol. 2009 May 20;10(5):R53. doi: 10.1186/gb-2009-10-5-r53 (PMC2718519; doi:10.1186/gb-2009-10-5-r53)
Supplement: Additional data file 3 — A PDF file showing maximum likelihood analysis of SNPs, listing loci where omega (dN/dS) is significantly greater than 1. [file gb-2009-10-5-r53-S3.pdf]

Additional data file 3. Results of Maximum Likelihood analysis of all 14 chromosomes

For each chromosome, compared
(i) Me49 with VEG,
(ii) Me49 with TgCkUg2,
(iii) VEG with TgCkUg2.

Pairwise comparisons, where omega (dN/dS) is significantly greater than 1.

Genomic Location	Strain1	Strain2	Gene	Product description	Omega	Kappa	t	x	^x	NxdN	SxdS	P
Ia: 1,403,087 - 1,410,897 (-)	Me49	TgCkUg2	83.m02145	ROP7	infinity	2.432	0.006	6	6.0	5.975	0.000	0.025<P<0.050
III: 25,847 - 26,506 (-)	Me49	VEG	64.m00739	hypothetical protein	infinity	11.339	0.028	5	5.1	5.058	0.019	0.025<P<0.050
III: 381,515 - 382,947 (-)	Me49	VEG	52.m03454	hypothetical protein	5.654	2.785	0.036	13	13.1	12.178	1.022	0.025<P<0.050
III: 813,521 - 817,895 (+)	Me49	VEG	52.m02683	hypothetical protein	3.004	1.204	0.323	74	81.8	71.510	10.296	0.000<P<0.005
III: 1,087,221 - 1,087,724 (-)	Me49	VEG	52.m03445	conserved hypothetical protein	infinity	11.214	0.030	5	5.1	5.052	0.037	0.025<P<0.050
III: 1,478,244 - 1,485,871 (+)	Me49	VEG	52.m02694	hypothetical protein	1.785	3.470	0.041	66	66.9	52.683	14.187	0.025<P<0.050
III: 1,899,140 - 1,900,667 (+)	Me49	VEG	52.m00002	GRA8	3.238	2.391	0.061	16	16.4	13.294	3.060	0.025<P<0.050
IV: 339,190 - 340,818 (-)	Me49	VEG	641.m03163	hypothetical protein	3.071	0.621	0.071	37	38.5	31.419	7.149	0.000<P<0.005
IV: 403,574 - 405,865 (-)	Me49	VEG	641.m01562	bradyzoite-specific surface protein	2.901	1.843	0.188	71	74.9	65.550	9.268	0.000<P<0.005
IV: 940,527 - 951,403 (+)	VEG	TgCkUg2	641.m01516	hypothetical protein	infinity	2.095	0.006	10	10.0	9.928	0.000	0.005<P<0.010
IV: 2,471,604 - 2,473,019 (+)	Me49	VEG	48.m00093	hypothetical protein	2.281	1.612	0.101	46	47.5	39.185	8.273	0.025<P<0.050
V: 243,458 - 246,171 (-)	Me49	VEG	39.m00623	proline-rich protein	3.955	3.380	0.062	32	32.7	28.693	4.062	0.000<P<0.005
V: 243,458 - 246,171 (-)	Me49	TgCkUg2	39.m00623	proline-rich protein	infinity	12.546	0.013	7	7.0	6.997	0.059	0.010<P<0.025
V: 941,849 - 942,772 (+)	Me49	VEG	31.m01829	hypothetical protein	infinity	2.788	0.030	9	9.1	9.038	0.031	0.005<P<0.010

Genomic Location	Strain1	Strain2	Gene	Product description	Omega	Kappa	t	x	^x	NxdN	SxdS	P
V: 1,291,001 - 1,292,655 (-)	Me49	TgCkUg2	31.m01816	iron-sulfur cluster assembly accessory protein	infinity	1.262	0.016	8	8.1	8.041	0.048	0.010<P<0.025
V: 1,927,848 - 1,928,810 (+)	Me49	VEG	76.m01639	hypothetical protein	infinity	12.644	0.019	6	6.1	6.024	0.036	0.010<P<0.025
V: 1,991,460 - 1,993,181 (-)	Me49	VEG	76.m01634	hypothetical protein	infinity	infinity	0.022	5	5.1	5.053	0.024	0.025<P<0.050
V: 3,018,505 - 3,027,457 (+)	Me49	TgCkUg2	76.m01544	hypothetical protein	infinity	1.163	0.003	9	9.1	9.263	0.000	0.010<P<0.025
VI: 1,898,443 - 1,907,404 (-)	Me49	VEG	49.m03279	hypothetical protein	infinity	3.226	0.003	7	7.1	6.805	0.000	0.010<P<0.025
VI: 2,460,809 - 2,469,031 (-)	Me49	VEG	49.m03334	BRCA2 repeat-containing protein	6.401	4.626	0.005	13	13.2	11.801	1.142	0.010<P<0.025
VI: 2,873,689 - 2,875,327 (-)	Me49	VEG	49.m03376	hypothetical protein	infinity	2.073	0.064	8	8.2	8.211	0.031	0.025<P<0.050
VI: 2,873,689 - 2,875,327 (-)	VEG	TgCkUg2	49.m03376	hypothetical protein	infinity	1.551	0.056	7	7.2	7.155	0.029	0.025<P<0.050
VI: 2,926,773 - 2,929,809 (-)	VEG	TgCkUg2	49.m03382	hypothetical protein	infinity	14.508	0.015	7	7.1	6.990	0.048	0.010<P<0.025
VI: 3,402,642 - 3,409,705 (+)	VEG	TgCkUg2	49.m03431	hypothetical protein	infinity	3.547	0.006	8	8.0	7.904	0.000	0.010<P<0.025
VIIa: 1,745,401 - 1,749,423 (-)	Me49	VEG	20.m03866	hypothetical protein	infinity	11.816	0.016	6	6.1	5.988	0.041	0.010<P<0.025
VIIa: 2,567,900 - 2,570,402 (+)	Me49	VEG	20.m00005	GRA7	5.150	1.108	0.066	16	16.3	15.323	1.006	0.025<P<0.050
VIIa: 2,589,211 - 2,595,538 (+)	Me49	VEG	20.m03783	ATP-dependent Clp protease proteolytic subunit	5.783	5.691	0.037	11	11.2	10.170	1.018	0.025<P<0.050
VIIa: 3,945,304 - 3,950,401 (-)	Me49	VEG	20.m03985	megakaryocyte stimulating factor	3.019	1.375	0.108	128	133.7	118.588	15.245	0.000<P<0.005
VIIb: 481,603 - 484,899 (+)	Me49	VEG	55.m08200	hypothetical protein	6.623	3.524	0.014	15	15.1	14.172	1.044	0.010<P<0.025
VIIb: 1,544,716 - 1,546,334 (-)	Me49	VEG	55.m04955	hypothetical protein	infinity	2.001	0.103	13	13.5	13.437	0.055	0.000<P<0.005
VIII: 5,386,572 - 5,387,878 (-)	Me49	TgCkUg2	59.m07776	conserved hypothetical protein	infinity	4.882	0.035	6	6.1	6.059	0.035	0.025<P<0.050
VIII: 6,692,379 - 6,704,572 (+)	Me49	TgCkUg2	59.m03361	200 kDa antigen p200-related	4.325	3.437	0.007	21	21.1	19.042	1.893	0.010<P<0.025
VIII: 6,882,242 - 6,886,119 (+)	Me49	VEG	8.m00192	hypothetical protein	infinity	3.765	0.006	6	6.0	6.053	0.000	0.025<P<0.050

Genomic Location	Strain1	Strain2	Gene	Product description	Omega	Kappa	t	x	^x	NxdN	SxdS	P
IX: 1,785,965 - 1,789,313 (+)	Me49	VEG	57.m00014	co-chaperone GrpE	infinity	4.131	0.013	6	6.1	5.968	0.044	0.025<P<0.050
IX: 3,576,654 - 3,579,197 (-)	Me49	VEG	80.m03974	hypothetical protein	infinity	5.218	0.011	9	9.1	8.935	0.082	0.005<P<0.010
IX: 6,373,197 - 6,374,206 (-)	Me49	VEG	541.m01251	hypothetical protein	infinity	3.415	0.048	8	8.2	8.131	0.029	0.010<P<0.025
X: 61,766 - 62,728 (-)	Me49	TgCkUg2	42.m07434	hypothetical protein	infinity	2.437	0.047	8	8.1	8.090	0.030	0.010<P<0.025
X: 894,498 - 898,809 (-)	VEG	TgCkUg2	42.m03570	lytB domain-containing protein	infinity	2.809	0.007	5	5.1	4.960	0.000	0.025<P<0.050
X: 991,698 - 993,865 (-)	VEG	TgCkUg2	42.m00013	GRA3	infinity	1.855	0.041	9	9.1	9.114	0.036	0.010<P<0.025
X: 3,621,460 - 3,621,966 (-)	Me49	VEG	42.m07410	hypothetical protein	infinity	1.618	0.043	7	7.2	7.140	0.033	0.010<P<0.025
X: 3,939,372 - 3,940,433 (-)	Me49	VEG	28.m00550	hypothetical protein	6.691	infinity	0.035	12	12.2	11.274	0.990	0.010<P<0.025
X: 4,579,251 - 4,584,450 (+)	Me49	VEG	46.m00012	hypothetical protein	10.316	3.901	0.048	21	21.4	20.395	0.988	0.000<P<0.005
X: 5,503,600 - 5,505,578 (-)	Me49	VEG	46.m01717	hypothetical protein	infinity	2.264	0.028	6	6.1	6.030	0.018	0.025<P<0.050
X: 5,689,815 - 5,698,810 (-)	VEG	TgCkUg2	46.m02909	hypothetical protein	infinity	7.159	0.004	5	4.9	5.092	0.000	0.025<P<0.050
X: 5,982,119 - 5,986,510 (+)	Me49	VEG	33.m01261	hypothetical protein	4.489	1.440	0.016	22	22.1	20.227	2.032	0.010<P<0.025
X: 7,213,002 - 7,216,378 (-)	Me49	VEG	63.m00002	GRA6	5.838	2.605	0.059	13	13.3	12.288	0.998	0.025<P<0.050
XII: 137,376 - 141,329 (+)	Me49	TgCkUg2	145.m00337	hypothetical protein	6.527	1.663	0.009	12	12.1	10.944	1.025	0.010<P<0.025
XII: 517,164 - 523,223 (+)	Me49	TgCkUg2	551.m00237	hypothetical protein	infinity	infinity	0.004	4	4.0	4.018	0.000	0.025<P<0.050

Key:

omega	dN/dS ratio.
kappa	transversion/transition rate ratio.
t	Sequence distance (codon substitutions per codon).
x	Observed nucleotide substitutions (i.e., number of SNPs detected between II and III).
^x	Estimated nucleotide substitutions, after correction for multiple hits.
NxdN	Estimated number of non-synonymous nucleotide substitutions.
SxdS	Estimated number of synonymous nucleotide substitutions.
P	Outcome of LRT analysis showing how significantly different omega is from 1.

Procedure applied to each chromosome, in more detail:
==

1) Protein coding DNA extracted from Me49 chromosome.

2) Equivalent DNA sequences for VEG, generated from Me49 using Me49 vs VEG SNP data.

3) Equivalent DNA sequences for TgCkUg2, generated from (i) Me49, and (ii) VEG (which is itself generated from Me49).

4) For each gene, compared:
   (i) Me49 coding sequence with equivalent VEG sequence (generated from Me49),
   (ii) Me49 coding sequence with equivalent TgCkUg2 (generated from Me49),
   (iii) VEG coding sequence with equivalent TgCkUg2 (generated from VEG).

   Each comparison involved (i) maximum likelihood (ML) pairwise comparison to estimate dN/dS (omega), (ii) an equivalent ML comparison, but with omega fixed at 1, (iii) a likelihood ratio test (LRT) to assess whether the estimated omega was significantly greater than 1.

   In all cases, ML analysis was carried out according to Goldman and Yang (1994) as implemented in the codeml program within the Paml (version 4b) package. Model assumed was:
   - All 61 codon frequencies calculated separately (F61).
   - Kappa (transition/transversion rate ratio) estimated.
